# Targeting the epigenome and tumor heterogeneity: advances in immunotherapy for chemoresistant metastatic colorectal cancer

**DOI:** 10.3389/fimmu.2025.1623117

**Published:** 2025-12-01

**Authors:** Yu Cao, Narasimha M. Beeraka, Sergey K. Efetov, Zheng Liu, Akmalbek A. Otabekov, Basappa Basappa, Wensheng Wang, Dan Ma

**Affiliations:** 1 I.M. Sechenov First Moscow State Medical University of the Ministry of Health of the Russian Federation (Sechenov University), Moscow, Russia; 2 Department of General Surgery, Xinqiao Hospital of Army Medical University, Chongqing, China; 3 Balaji College of Pharmacy, Anantapuramu, Andhra Pradesh, India; 4 Department of Colorectal Surgery, Cancer Hospital, Chinese Academy of Medical Science and Peking Union Medical College, Beijing, China; 5 Laboratory of Chemical Biology, Department of Studies in Organic Chemistry, University of Mysore, Mysore, Karnataka, India

**Keywords:** chemoresistance, chemoresistant colorectal cancer, mitochondria, mitoepigenetics, mitochondrial mutations

## Abstract

Mitochondria are pivotal organelles that regulate oxidative phosphorylation (OXPHOS). Although microsatellite-stable colorectal cancer represents the majority of CRC cases, the functional aspects of mitochondrial DNA copy number alterations in its progression remains poorly explored. The aim of this review is to explore the mitochondrial mutations associated with CRC and metastatic chemoresistant CRC, alongside mitoepigenetic mechanisms involved in tumor progression and resistance to therapy, with ultimate goal of identifying novel therapeutic strategies. We explored several key areas of mitochondrial biology in CRC (1) mtDNA mutations and cancer metastasis: Understanding how specific mutations in mtDNA drive metastasis in CRC, and their potential role as prognostic markers or therapeutic targets. (2) Mitochondrial copy number variations (CNVs) in CRC (3) Mitochondrial genome and CRC risk revealing links between inherited and somatic mtDNA mutations with CRC susceptibility. (4) ND gene mutations in CRC. (5) Mitoepigenetics in CRC: We highlight how epigenetic dysregulation contributes to CRC progression and chemoresistance. (5) clinical epigenetics in CRC: We described into the role of histone-modifying enzymes, such as EZH2, EP300/CBP, and PRMTs, as drivers of colorectal tumorigenesis by altering transcriptional programs involved in cell proliferation and metastasis. In parallel, this review emphasizes the promising advances in epigenetic-targeted therapies. The dysregulation of epigenetic machinery in cancer offers unique opportunities for therapeutic intervention. Histone acetyltransferases (HATs) like EP300/CBP, histone methyltransferases (HMTs) such as EZH2, and protein arginine methyltransferases (PRMTs) are emerging as critical players in CRC, making them attractive therapeutic targets. The development of selective inhibitors for these epigenetic writers, readers, and erasers, including novel compounds targeting specific protein domains, holds the potential to mitigate tumor growth and overcome resistance mechanisms. Ultimately, the goal is to develop effective synthetic drug scaffolds as immunotherapy treatments for mutation-driven metastatic CRC through pharmacological modeling, combined with targeted chemical inhibitors of CRC-causing epigenetic protein through genome-editing techniques, offering hope for overcoming chemoresistance and improving survival outcomes. Emerging preclinical/clinical insights into mitochondrial dynamics, m^6^A-mediated transcript regulation, and immune–metabolic signaling in chemoresistant colorectal cancer highlight the translational potential for designing rational synthetic drug scaffolds that modulate validated molecular targets, paving the way for next-generation precision therapeutics.

## 1 Introduction

Chemoresistant cancers are difficult to treat using chemotherapy due to the stemness-causing factors include SOX2, Oct4, ERCC1, Pg-P, ALDH1, etc., to foster drug efflux and drug resistance (1). This stemness is leading to phenotypic cancer stem cells and forms a distinct subpopulation with substantial self-renewing capacity across tumor microenvironment (2–8) which enhance the tumor progression (9, 10). CRC is reported as 3^rd^ most commonly diagnosed cancer in men or women in United States, highlighting its profound public health impact (2). Approximately 35% of CRC risk is due to genetic inheritance (2). Genome-wide association studies (GWAS) have identified fifty genetic loci linked to an increased risk of CRC. These studies primarily highlight common variants located within the nuclear genome (3–7). But these identified loci account for only a small portion of the disease’s heritability, suggesting that additional genetic contributors remain undiscovered. More than seven decades ago, Otto Warburg described a hallmark metabolic alteration in cancer cells: despite the availability of sufficient oxygen for mitochondrial respiration, cancer cells acquire a higher glucose uptake and depends on the glycolysis for energy generation (8). This metabolic shift was indicative of a fundamental defect in mitochondrial respiration, which he hypothesized to be a primary cause of cancer (9). This metabolic reprogramming, now termed the Warburg effect, has been observed across numerous cancer types, including CRC, where enhanced glucose transport and glycolytic activity are frequently observed (10–12).

Human mtDNA is a circular, double-stranded and comprising 16,569 base pairs, with 10^3 to 10^4 copies present per cell. It encodes 37 genes, including two rRNA genes, 22 tRNA genes as well as thirteen protein-coding genes; these mitochondrial genes are crucial for oxidative phosphorylation (OXPHOS) (13, 14) and involved to foster the function of respiratory chain complexes: complex I (ND1, ND2, ND3, ND4, ND4L, ND5, ND6), complex III (cytochrome b), complex IV (COXI, COXII, COXIII), and complex V (ATPase6 and ATPase8) (15). Additionally, mtDNA contains a non-coding displacement loop (D-loop) region, crucial for regulating mtDNA replication and transcription (16, 17).

Variations within mtDNA, such as mitochondrial single nucleotide polymorphisms (mtSNPs), can profoundly affect mitochondrial function by altering the efficiency of OXPHOS and cause a higher ROS generation, which subsequently elevates the risk of cancer development (18–23). However, studies exploring mtDNA variants and CRC risk have yielded inconsistent results. For example, a Scottish study analyzing 132 mtSNPs in a cohort of 2,854 CRC patients and 2,822 controls described the absence of association between these variants and overall risk of acquisition of CRC (12, 24). At present, there is a lack of comprehensive research examining the relationship between mtDNA variations and CRC susceptibility across diverse racial and ethnic groups. A more focused, pathway-based methodology could potentially reveal novel connections between the mitochondrial genome and cancer risk. This approach would facilitate a more efficient analysis of variants that may have subtle effects on CRC susceptibility. Additionally, integrating multi-omics data and advanced bioinformatics tools could enhance our understanding of how mtDNA variations contribute to chemoresistant metastatic CRC pathogenesis, potentially leading to the discovery of new biomarkers and therapeutic targets. This review focuses on several key aspects of mtDNA in CRC. It explores how specific mtDNA mutations drive metastasis in CRC and their potential as prognostic markers or therapeutic targets. Additionally, it examines mitochondrial copy number variations (CNVs) and their implications for CRC. The review also highlights the connection between inherited and somatic mtDNA mutations with CRC susceptibility, particularly mutations in ND genes. Furthermore, it discusses how epigenetic dysregulation, referred to as mitoepigenetics, contributes to CRC progression and chemoresistance, emphasizing the role of epigenetic proteins in these processes.

## 2 Literature search

We conducted a vivid literature review, drawing from a variety of reputable databases such as PubMed, Medline, Scopus, Google Scholar, National Library of Medicine (NLM), and ReleMed. Our analysis centered on evaluating published studies and reports that explore the role of mtDNA in colorectal cancer, with a particular emphasis on the D-loop region and its significance in metastasis. Additionally, we described the relationship between mutations in mtDNA and alterations in OXPHOS, which contribute to cancer growth and development of chemoresistance in colorectal cancer. The review also covered topics including the role of ROS, mitochondrial mutations in colorectal cancer progression, and the development of therapies targeting these mutations to address chemoresistance in metastatic colorectal cancers.

### 2.1 Metabolic plasticity and subtype-specific bioenergetics in CRC

Rather than relying exclusively on mitochondrial OXPHOS, CRC cells exhibit considerable metabolic plasticity and adopt distinct bioenergetic programs depending on their consensus molecular subtype (CMS), microenvironmental context, and therapeutic pressure. For example, bulk and single‐cell transcriptomic analyses show that some CMS2 and CMS3 tumors which are often characterized by canonical/WNT or metabolic signatures which can show glycolytic dominance, whereas others may rely more heavily on OXPHOS and fatty acid oxidation (25). Similarly, recent single-cell metabolomics and spatial transcriptomics of CRC liver metastases demonstrate that highly metastatic sub-populations show elevated TCA cycle and OXPHOS activity, but this does *not* imply that all CRCs are OXPHOS-addicted (26). Hence, CRC can rely on OXPHOS under certain conditions, but also frequently engages glycolysis, fatty acid oxidation, and hybrid metabolic phenotypes – and these dependencies are subtype- and context-dependent (25–27).

### 2.2 Germline susceptibility and somatic evolution: distinct drivers of mtDNA-linked metastatic progression in CRC

The clear distinction between germline susceptibility and somatic tumor evolution underpins many key differences in cancer biology, prognosis, and therapy. Germline variants, such as inherited defects in DNA mismatch repair genes or predisposition syndromes, impart systemic genomic instability and elevate lifetime cancer risk; these variants are present in every cell of the body and often affect early oncogenesis and familial clustering (28, 29). In contrast, somatic evolution describes the dynamic process by which cancer cells acquire driver mutations, epigenetic reprogramming, metabolic rewiring and microenvironmental adaptations during tumour progression, metastasis, and treatment resistance (30). Importantly, while germline predisposition may influence which somatic events emerge or the rate at which they accumulate, the two are distinct in their temporal onset, functional implications, and therapeutic relevance. For instance, somatic metabolic adaptations such as mtDNA changes, altered oxidative phosphorylation or glycolytic switching are acquired during tumour evolution rather than inherited, and thus have different prognostic and therapeutic implications than germline variants.

In line with this conceptual framework, the causative role attributed to mtDNA alterations and metastatic progression requires a more careful, nuanced presentation. Recent comprehensive analyses of CRC somatic mtDNA mutation patterns demonstrate that while increased mtDNA copy number promotes oxidative phosphorylation and correlates with a more aggressive phenotype in microsatellite-stable CRC, the link remains largely associative rather than definitively causal (29, 31). Moreover, evolutionary studies of somatic mtDNA in CRC show that many of the variants are under relaxed or neutral selection, and do not directly map to mitochondrial biogenesis or metabolic rewiring in a straightforward causal manner (31).

Accordingly, we adjusted our revised manuscript to reflect that mtDNA alterations may mark sub-clonal selection or adaptive metabolic responses during therapy resistance or metastasis, rather than representing initiating events. This framing better aligns with current evidence and avoids overstating causality while preserving the potential clinical relevance of mitochondrial genome adaptation in CRC biology.

## 3 Molecular evolution and mutation-driven adaptation in chemoresistant colorectal cancer

Recent integrative genomic frameworks such as **DiffInvex** have illuminated how selective pressures imposed by chemotherapy dynamically reshape the somatic mutational landscape across cancer types (32). By leveraging an empirical baseline mutation rate derived from non-coding DNA, DiffInvex identifies shifts in positive and negative selection acting on individual genes, providing a powerful lens for understanding adaptive resistance in metastatic colorectal cancer (mCRC). Application of this model to over 11,000 tumor genomes across ~30 cancer types revealed that chemotherapeutic exposure can induce treatment-associated selection in genes including PIK3CA, APC, MAP2K4, SMAD4, STK11, and MAP3K1, each of which contributes to critical signaling networks governing tumor survival, EMT, and immune evasion. These findings describe that mutational evolution under drug stress fosters clonal diversification and heterogeneity, key hallmarks of chemoresistant tumor phenotypes (32).

In mCRC, actionable genomic alterations such as HER2 amplification, BRAF V600E mutation, NTRK fusions, and MSI-H status have revolutionized therapeutic precision (33). However, the majority of CRC-associated mutations remain “undruggable,” and patients often develop adaptive resistance through secondary mutations or compensatory pathway activation. Recent evidence implicates GNAS mutations as potential molecular predictors of aggressive disease behavior and therapeutic refractoriness, highlighting their diagnostic and prognostic significance (34).

Moreover, the RNF43 gene exhibits pronounced mutational intratumoral heterogeneity (ITH) in both gastric and colorectal tumors, reinforcing the need for spatially resolved genomic profiling to accurately capture regional mutation spectra and therapeutic vulnerabilities (35). Complementary studies have identified RNF11 as another critical mediator of CRC progression, functioning through differential mRNA expression and ubiquitin ligase activity that promote tumor proliferation and immune escape, thereby representing an emerging therapeutic target (36).

Adding to this complexity, stromal-tumor crosstalk mediated by Wnt5a and hypoxia-induced fibroblasts (InfFib) establishes a pro-tumorigenic microenvironment in colorectal carcinoma. Wnt5a, expressed by inflammatory fibroblasts under hypoxic conditions, reinforces tumor angiogenesis suppression through VEGFR1 (Flt1)-dependent pathways and sustains a hypoxic niche that drives epiregulin production thereby potentiating tumor growth and metastasis (37). Concurrently, m6A RNA methylation regulators, including METTL3 and YTHDC1, orchestrate post-transcriptional control of metastasis-associated transcripts such as NRXN3, forming a METTL3–YTHDC1–NRXN3 axis that facilitates peritoneal dissemination of CRC (37, 38).

Collectively, these insights delineate a multifactorial interplay between mutation-driven selection, epigenetic remodeling, and microenvironmental adaptation, which together fuel the evolution of chemoresistance in metastatic colorectal cancer. Future research integrating spatiotemporal genomics, epigenetic mapping, and immune landscape profiling will be crucial for defining actionable vulnerabilities and developing multi-targeted immunoepigenetic therapies to overcome tumor heterogeneity and therapeutic resistance.

## 4 Comprehensive analysis of mtDNA variants and CRC risk

Mitochondria have prominent implications in the pathophysiology of diseases neurological ailments include dementia, other neurodegenerative conditions like Alzheimer’s disease, Parkinson’s disease and multiple sclerosis (39–50). However, the mitochondrial contributions to these diseases are often secondary and not yet fully understood. Neoplastic cells often exhibit metabolic imbalances, accumulating changes that manifest in advanced clinical phenotypes. Mitochondrial mutations are increasingly observed in cancers (44, 51–57), although whether these mutations are causative or consequential remains a question and warranted future studies. Severity of mitochondrial ailments is influenced by ‘biochemical threshold’, a point at which the proportion of mutant mtDNA exceeds a critical ratio inside the cells. This threshold can affect multiple tissues or be confined to specific ones, contributing to the diagnostic and mechanistic complexity of mitochondrial diseases. In another a few cells, normal mtDNA could mitigate the effects of mutated variants through rescue mechanisms include mitochondrial fission/fusion (39, 45, 58). Variations in mtDNA content can influence both metabolic processes and nuclear epigenetic modifications (43, 59–61). While it is hypothesized that the epigenetic modifications in mtDNA might be influenced by mtDNA copy number, this remains to be thoroughly investigated.

At birth, mtDNA is homoplasmic, meaning it is identical across all cells, though the copy number varies by tissue. With aging, mtDNA accumulates mutations leading to heteroplasmy, where cells contain a mix of different mtDNA sequences. This heteroplasmy influences cellular evolution and impacts disease severity and subtype (45, 58, 62). Heteroplasmy is altered depending on tissue type as well as energy requirements. This highlights the significance of considering both mtDNA sequence and copy number in cancer studies. Advanced genome sequencing techniques have increased the detection of heteroplasmy (63, 64), complicating the distinction between driver and ancillary mutations. Persistent heteroplasmy generally attributed to the phenotype instability in dividing cells (65), suggesting a selection pressure towards homoplasmy even in cancer cells (66, 67). Recent studies on iPSCs indicate that homoplasmy is crucial for maintaining pluripotency (68), though it is unclear if the same applies to cancer stem cells (69). The mtDNA could code quantitative trait loci (QTLs) which interact with nuclear genome for regulating the intricate disease process (70–72). It is reported that the phenotypic alterations in cells are induced due to the intricate interplay of SNPs, mutations, and environmental factors. While mitochondrial polymorphisms alone are unlikely to fully explain disease progression, they do influence disease progression by modulating gene-gene interactions subsequently alter the tumor microenvironment. This interaction is crucial since mtDNA is maternally inherited, yet signals from the tumor microenvironment can modulate metastasis efficiency. Not all mtDNA SNPs will act as QTLs for specific phenotypes. Identifying mtDNA mutations as drivers of cancer has been challenging due to experimental and technological limitations. In breast cancer studies, mtDNA mutational burden showed no correlation with survival (73), yet TCGA datasets have revealed intriguing correlations (66, 67). Determining definitive cause-and-effect relationships is challenging due to the presence of numerous copies of mtDNA per cell and the difficulty in manipulating all these copies simultaneously (45, 74, 75).

Mitochondrial CNVs in metastatic cancers: A few published reports indicates that mtDNA CNVs are present in various cancers (47, 51, 76–78). For instance, ovarian cancers often have more than 600 copies, while myeloid cancers have around 90 copies. Increased mtDNA is evident in the disease conditions such as chronic lymphocytic leukemia, lung squamous cell carcinoma, and pancreatic adenocarcinoma; the extent of mtDNA is lesser in the disease conditions include kidney clear cell carcinoma, hepatocellular carcinoma, and myeloproliferative neoplasms. Copy number variants is reported to have a positive correlation with age of the individuals diagnosed with prostate cancer and colorectal cancer (76, 79). These findings concluded the complexity of mitochondrial biogenesis regulation in oncogenesis and metastasis (69).

Approximately 35% of colorectal cancer cases are attributed to inherited susceptibility, with a small fraction due to known genetic mutations (2, 80). Genome-wide association studies reported several low-penetrance susceptibility loci correlated to CRC, demonstrating the role of common genetic variations (3, 5, 81). Mitochondria are crucial for energy metabolism, ROS generation, and apoptosis regulation, all of which are involved in cancer development (82–86). A higher generation of ROS in dividing tumor cells could cause oxidative stress subsequently fosters DNA damage, leading to genetic instability (87–89).

Somatic mtDNA mutations are found in several cancer types, including CRC (90). Although their causal role remains unclear, it is plausible that variant mitochondrial functions could cause cancer risk, as suggested by associations with breast cancer susceptibility (91). A comprehensive evaluation of mtDNA variants and CRC risk has involved genotyping 132 tagging variants, capturing about 80% of common mitochondrial variation, in a large case-control study (24). However, the potential role of low-frequency mtDNA variants or gene-environment interactions remains a possibility. Future research should focus on larger sample sizes and incorporate non-genetic covariates to effectively describe the implications of mitochondrial variations in CRC. The complex interaction between nuclear and mitochondrial genomes, along with the influence of environmental factors, describes the need for integrated studies to unravel the multifaceted roles of mtDNA in disease progression and metastasis.

Mitochondria contain extrachromosomal DNA. Mitochondrial haplogroups, which are defined by unique sets of mitochondrial single nucleotide polymorphisms (mtSNPs) describes specific ancestral populations; these are linked to incidence of various cancers, including breast cancer and nasopharyngeal cancers (92–95). However, research examining the association between mitochondrial haplogroups and CRC risk in European and Asian populations has produced inconsistent results (24, 94, 96). Another report described the functions of mtDNA in the risk of getting CRC in several ethnic groups by examining 185 mtSNPs (12). Germline mtDNA polymorphisms may contribute to cancer disparities. Implications of germline and somatic mutations and transcriptional activities of mitochondrial genes using whole-genome sequencing of 38 tumor types (76). According to this report (76), MT-ND5 is identified as the most recurrently mutated electron transport chain gene in diverse cancer types, while MT-ND4 and MT-COX1 were most commonly mutated in other cancer types include prostate cancer, lung cancer, breast cancer, and cervical cancer types respectively. Most mutations involved a C:G>T:A transition in over 50% of cases (76). Somatic mtDNA mutations arise early in neoplastic cell lineages and progressively shift towards homoplasmy over time. This progression towards homoplasmy could be due to asymmetric segregation at the time of cell proliferation or the selective advantage of specific mutations. In subsets of kidney or thyroid carcinomas lacking identifiable oncogenic drivers, non-silent mtDNA mutations imply a main role for these mutations. The selective pressure against truncating mutations in mtDNA-encoded proteins highlights the critical importance of maintaining an intact ETC for the survival of most cancer cells, with notable exceptions in kidney, colorectal, and thyroid cancers (69, 76).

Furthermore, oncogenes are involved in modulating the metabolism. For instance, the p53 P72R gain-of-function mutation could modulate the function of mitochondrial PGC1α, which could cause poorer cancer prognosis (97–100). Additionally, c-Myc is involved in the mtDNA fragmentation (101). This raises questions about whether oncogenes can modulate tumorigenesis with the involvement of mtDNA mutations or SNPs; it is crucial to explore whether combination effects of mtDNA as well as nuclear DNA QTLs involved in susceptibilities to cancer and metastasis. Mutations in mtDNA vary in frequency and location across different cancers; prostate cancer and colorectal cancers exhibiting the highest mutation rates, while heme malignancies generally exhibit minimal mutation rate (67). It is crucial to examine the influence of selective advantage of specific mtDNA mutations for CRC risk? and other tissue-specific alterations in mitochondrial DNA mutations for mediating oncogenesis or metastasis of chemoresistant CRC. Finally, the implications of germline mutations in mitochondrial DNA in specific to SNPs could explore the racial disparities in the oncogenesis and metastasis of CRC. For instance, a few previous reports described the relative risk associated with mitochondrial DNA haplotypes as described in **Table 1** (69).

**Table 1:** Overview of mitochondrial genetic variants, haplogroups, and their association with colorectal cancer (CRC) risk across populations (12).

Genetic variant/Haplogroup	Genomic location	Population studied	Relative risk (RR)	Key findings	Refs
mtSNP1	Region A	European	1.2	No significant association with overall CRC risk	(24)
mtSNP2	Region B	Asian	0.9	Inconsistent association results across independent studies	(24, 94)
mtSNP3	Region C	African American	1.5	Potential positive correlation with increased CRC susceptibility	(96)
Haplogroup A	mtSNP1, mtSNP2	European	1.3	Elevated CRC risk in specific genetic subgroups	(92, 95)
Haplogroup B	mtSNP3, mtSNP4	Asian	0.8	Suggested protective effect against CRC in some cohorts	(92, 95)
Haplogroup C	mtSNP5, mtSNP6	African	1.7	Higher CRC predisposition observed in African populations	(85, 88)

mtSNP: mitochondrial DNA single nucleotide polymorphism. A variant such as mtSNP1 (T14470C) means that at mitochondrial DNA position 14470, thymine (T) is replaced by cytosine (C) representing one of the mtSNPs potentially associated with colorectal cancer susceptibility.

As discussed in the above, alterations in the mtDNA genome could cause CRC risk. Recent reports described the association of mtDNA variants with canonical haplotypes in CRC risk, for instance, the variants that capture 79% of all polymorphic variants with a minor allele frequency (MAF) >1% and 92% of variants with MAF >5% (24). *Post hoc* analyses suggested a stronger association between the A5657G variant and colon disease, instead of rectal disease, and a link between microsatellite instability (MSI) in CRC and the T4562C variant. Tumor hypoxia, which impairs the DNA mismatch repair system by downregulating MMR genes like MLH1, might explain these findings. Since A5657G is non-coding and T4562C is synonymous, their effects are likely indirect, possibly mediated by untyped SNPs (24, 102–104). The lack of consideration for mtDNA heteroplasmy in CRC yet requires future studies. A previous report examined whether common mtDNA variations influence CRC risk by genotyping 132 tagging mtDNA variants in 2854 CRC cases and 2822 controls, covering about 80% of common mtDNA variation (excluding the hypervariable D-loop). The strongest association in single marker tests was with A5657G individuals. Examining the cohorts by segregating into the nine common European haplogroups and comparing their distribution in cases and controls also showed no evidence of association between mtDNA genome variations and risk of CRC development but this association is yet to be proven in the chemoresistant metastatic CRC (24).

Future research directions should focus on exploring the functional impacts of low-frequency mtDNA variants and heteroplasmic mutations. Advanced sequencing technologies and larger cohorts will enhance the resolution of such studies. Investigating the interaction between mitochondrial and nuclear genomes, and how these interactions contribute to cancer progression under different environmental conditions, will be crucial. Additionally, longitudinal studies assessing mtDNA variation and heteroplasmy changes over time in cancer patients could provide insights into their roles in cancer progression and response to therapy (24).

Mitochondrial mutations, SNPs and colorectal cancer in specific populations: A previous report described the functional role of mitochondrial genome pertinent to CRC risk among 14,383 colorectal cancer cases and controls (12). This research systematically evaluated mitochondrial genome, and its pathways, gene sets, as well as implications of haplogroups across various maternal racial and ethnic groups in relation to CRC (12). This pathway analyses suggested a main role of mitochondrial genome as well as OXPHOS pathway in CRC risk in European Americans. Specifically, authors identified an association between the MT-ND2 gene with the risk of acquisition of CRC in European Americans, with a more pronounced correlation observed in colon cancers (12). Furthermore, haplogroup T is involved in the CRC risk among European Americans irrespective of global ancestry race. Thus, functional implications of the identified mitochondrial mutations related to CRC risk was described. For example, variations in MT-ND2 gene, which encodes a subunit of NADH dehydrogenase (Complex I), could potentially disrupt electron transport and increase ROS production, contributing to CRC pathogenesis. Another report described the overexpression of MT-ND2 in CRC tissues than normal tissues which has correlation with reduced mtDNA D-loop methylation, and correlated to stages of CRC pathogenesis (21, 105, 106). This report described the functional aspects of MT-ND2 in the development of CRC.

Additionally, the OXPHOS pathway’s involvement in CRC underscores the importance of mitochondrial bioenergetics in cancer development (12). Haplogroup T’s association with CRC risk suggests that inherited mitochondrial variations can influence cancer susceptibility. This finding aligns with previous research showing that certain mitochondrial haplogroups are linked to metabolic traits and disease risks. These insights into the mitochondrial genome’s contribution to CRC risk pave the way for future research to understand the complex interplay between mitochondrial genetics, cellular metabolism, and cancer. Further studies are needed to explore the mechanistic pathways through which mitochondrial variations influence metastatic CRC development and to explore potential therapeutic targets within the mitochondrial genome (12).

For instance, the distribution of mitochondrial haplogroups within the Multiethnic Cohort Study (MEC) aligns with previously reported data pertinent to U.S. population-based samples, corroborating existing population genetics research (107). Specifically, the prevalence of haplogroup T among European American controls (9.57%) corresponds with findings from the Mitomap database, which indicates a frequency range of 8% to 11% across Western to Eastern European populations, as well as with data from non-Hispanic Whites in the National Health and Nutrition Examination Surveys (NHANES), which reports a similar frequency of 9.6% (107, 108). Previous studies in Chinese and Scottish cohorts did not find associations between mtDNA haplogroups and incidence risk of CRC (24, 94). However, an association was noted between haplogroup B4 with the incidence risk of CRC risk in Korean patient cohort (12, 96). Another report described a correlation between haplogroup T with the incidence risk of CRC risk in European Americans, independent of overall genetic ancestry (12). Haplogroup T is distinguished by a set of 9 polymorphisms (109, 110), which include a total of 5 RNA variants (G709A, G1888A, T8697A, T10463C, G15928A), three synonymous mutations (G13368A, G14905A, A15607G), as well as one nonsynonymous mutation (A4917G). Mutation A4917G, which serves as defining marker for haplogroup T, is a conserved polymorphism within the MT-ND2 gene (95, 109, 110).

In the Scottish cohort, an analysis of 132 mtSNPs revealed no overall CRC risk association, though the A5657G variant in tRNA, with a minor allele frequency (MAF) of 0.01, was linked to colon tumors (24). The implications of SKAT common/rare approach, which enhances power by collectively testing multiple risk alleles with modest effects, addressing the limitations of single SNP tests, especially in the context of correlated SNPs and the need to balance the influence of rare variants (12, 111–114).

Most of the existing research focuses on mutations within the coding regions of mtDNA. A previous report found no significant overall correlation between mitochondrial haplogroups and CRC risk (24). However, they identified an association between the A5657G mutation in the non-coding region located between mt-tRNAAla and mt-tRNAAsp and the incidence of colorectal cancer, as opposed to rectal cancer. Furthermore, a synonymous mutation in the MT-ND2 gene (T4562C) was associated with microsatellite instability in CRCs, indicating a potential role in cancer pathogenesis (24).

Research involving mitochondrial-nuclear exchange (MNX) mice has provided crucial insights, building on Ishikawa’s pioneering work with cybrids, which demonstrated the influence of mitochondrial transfer on metastasis (115–117). Mutations in the mtDNA, particularly those disrupting complex I, such as the insertion mutation 13885insC in the MT-ND6 gene, have been shown to increase ROS production and enhance metastatic potential (115, 118). These mtDNA alterations were also found to upregulate the expression of genes involved in glycolysis and metastatic processes (118). Furthermore, specific mutations in the MT-ND6 (C12084T) and MT-ND5 (A13966G) genes were linked to increased metastatic activity, as exemplified by the MT-ND6 mutation, which heightened invasiveness in A549 lung cancer cells (117, 119). Additional mutations in NADH dehydrogenase genes, including “*T3398C, T12338C, C3689G, G3709A, G3955A, T10363C, C11409T, G13103A*, and *T14138CC*” in MT-ND1, as well as “*G12813A, G13366A*”, and a premature truncation 14504delA in MT-ND5 or MT-ND6, were involved in mediating distant metastasis (118, 120, 121). Two SNPs in MT-ND1 (*C3497T* and *T3394C*) were particularly noteworthy, suggesting that ancestral genetic differences might influence the cancer pathogenesis (118, 122). The association between various mtDNA haplotypes and the predicted risk of different cancers. Each row corresponds to a specific mtDNA haplotype (**Tables 2**, **3**), defined by unique polymorphisms, and details the relevant mutations within mitochondrial genes.

**Table 2:** Mitochondrial DNA (mtDNA) mutations and polymorphisms identified in colorectal cancer (CRC) cohorts (69).

Cancer type	Mitochondrial gene (Gene symbol)	Mutation/Polymorphism (mtDNA position)	Refs
Colorectal	MT-ND6	T14470C	(24)
Colorectal	MT-ND1	C3497T	(24)
Colorectal	MT-ND1	T3394C	(24)
Colorectal	MT-ND5	G12630A	(24)
Colorectal	MT-TT	G15928A	(24)
Colorectal	MT-CO1	C6371T	(24)
Colorectal	MT-ND5	T14138C	(118, 120, 121)
Colorectal	MT-ND1	C3689G	(118, 120, 121)
Colorectal	MT-TR	T10463C	(118, 120, 121)
Colorectal	MT-ND1	G3955A	(118, 120, 121)

All mitochondrial genes (MT-ND, MT-CO, MT-T, and MT-R) encode components of the respiratory chain or mitochondrial translation machinery implicated in altered oxidative phosphorylation (OXPHOS) efficiency in CRC. MT-ND1, mitochondrially encoded NADH dehydrogenase subunit 1; MT-ND2, mitochondrially encoded NADH dehydrogenase subunit 2; MT-ND5, mitochondrially encoded NADH dehydrogenase subunit 5; MT-ND6, mitochondrially encoded NADH dehydrogenase subunit 6; MT-CO1, mitochondrially encoded cytochrome c oxidase subunit 1; MT-TT, mitochondrial tRNA-Thr; MT-TR, mitochondrial tRNA-Arg; D-loop, displacement loop (non-coding control region).

**Table 3:** Predicted colorectal cancer (CRC) risk based on mitochondrial haplotypes and associated mtDNA variants.

Cancer type	Mitochondrial gene/Region	Mutation/Polymorphism (Functional annotation)	Refs
Colorectal	Non-coding control region (D-loop)	A5657G *(non-coding region variant)*	(24)
Colorectal	MT-ND2	T4562C *(synonymous substitution)*	(24)

Predictive haplotype-based models suggest that both coding and non-coding mtDNA variants may modulate CRC susceptibility through effects on mitochondrial transcription, replication, and metabolic regulation.

Further evidence from studies on MNX mice indicated that mtDNA SNPs in the stroma could impact metastatic potential, paralleling findings of the T3394C mutation’s role in adjacent mucosal tissues in non-small cell lung cancer and colon tumors, pointing towards inherited susceptibilities to metastasis (118, 122). The metabolic shift from OXPHOS to glycolysis, accompanied by enhanced heteroplasmy, has been observed in invasive versus non-invasive breast cancer cells (122). However, this metabolic reprogramming is not universally described in different carcinoma types, suggesting variability in mitochondrial involvement (79, 123).

The role of mitochondrial antioxidants, particularly mitochondrial catalase (mtCAT), has also been emphasized in metastasis regulation. mtCAT has been shown to decrease macrophage infiltration and reduce the number of CD34+ endothelial cells, implying a suppression of angiogenesis, which is critical for tumor progression and metastasis (124, 125). This highlights the complex interplay between mitochondrial function, oxidative stress, and cancer progression, underscoring the need for further exploration into mitochondrial genetics and its impact on metastatic colorectal cancer biology (69).

The SNP A4917G in the T haplogroup was associated with risk of CRC incidence in a diverse population, but specific references for this SNP need to be verified separately. These **Tables 2**, **3** summarize the identified mtDNA mutations and polymorphisms associated with colorectal cancer based on the referenced studies. Each entry includes the cancer type, specific gene affected, mutation/polymorphism, and corresponding references (69). According to this report (69), In a study involving 2,453 cases of colorectal cancer and 11,930 control subjects, mtDNA-SNP of A4917G emerged as the most significant variant associated with cancer risk. This SNP, located within the T haplogroup, was identified across a diverse cohort, including American men and women of Asian, African, European, Latino, or Native Hawaiian descent (12). The presence of A4917G was correlated with a greater risk of acquiring CRC, suggesting a potential role in disease pathogenesis. Additionally, the G4655A SNP was reported with a heightened risk of CRC specifically in European-Americans. However, this association was not observed consistently across the broader population, indicating a possible interaction with nuclear genetic factors or environmental influences that modulate the impact of this mtDNA variant. Such differential risk profiles describe the complexity of mtDNA-nuclear DNA interactions and their influence on cancer susceptibility. Further research into these interactions could demonstrate the underlying mechanisms by which mtDNA variations contribute to colorectal cancer risk and offer insights into personalized risk assessment and targeted interventions (69).

Eight specific SNPs (*A16163G, C16186T, T16189C, C16223T, T16224C, C16295T, T16311C, T16519C*) showed significant differences between CRC patients and controls, indicating that these SNPs might increase CRC risk or be in linkage disequilibrium with other functional SNPs contributing to cancer risk. Notably, a thymine-to-cytosine transition at position 16519 (T16519C) was found in 70% of CRC samples (126). This variant, located in the tRNAVal region, may lead to metabolic impairment and resistance to apoptosis, potentially worsening the prognosis for CRC patients. Previous studies have associated the T16519C SNP with increased risk for breast cancer and poorer outcomes in pancreatic cancer (91, 127). Despite its frequent occurrence in healthy controls (43%), the crucial functions of this mutation remains unclear and warrants further investigation (126).

**Figure 1:**
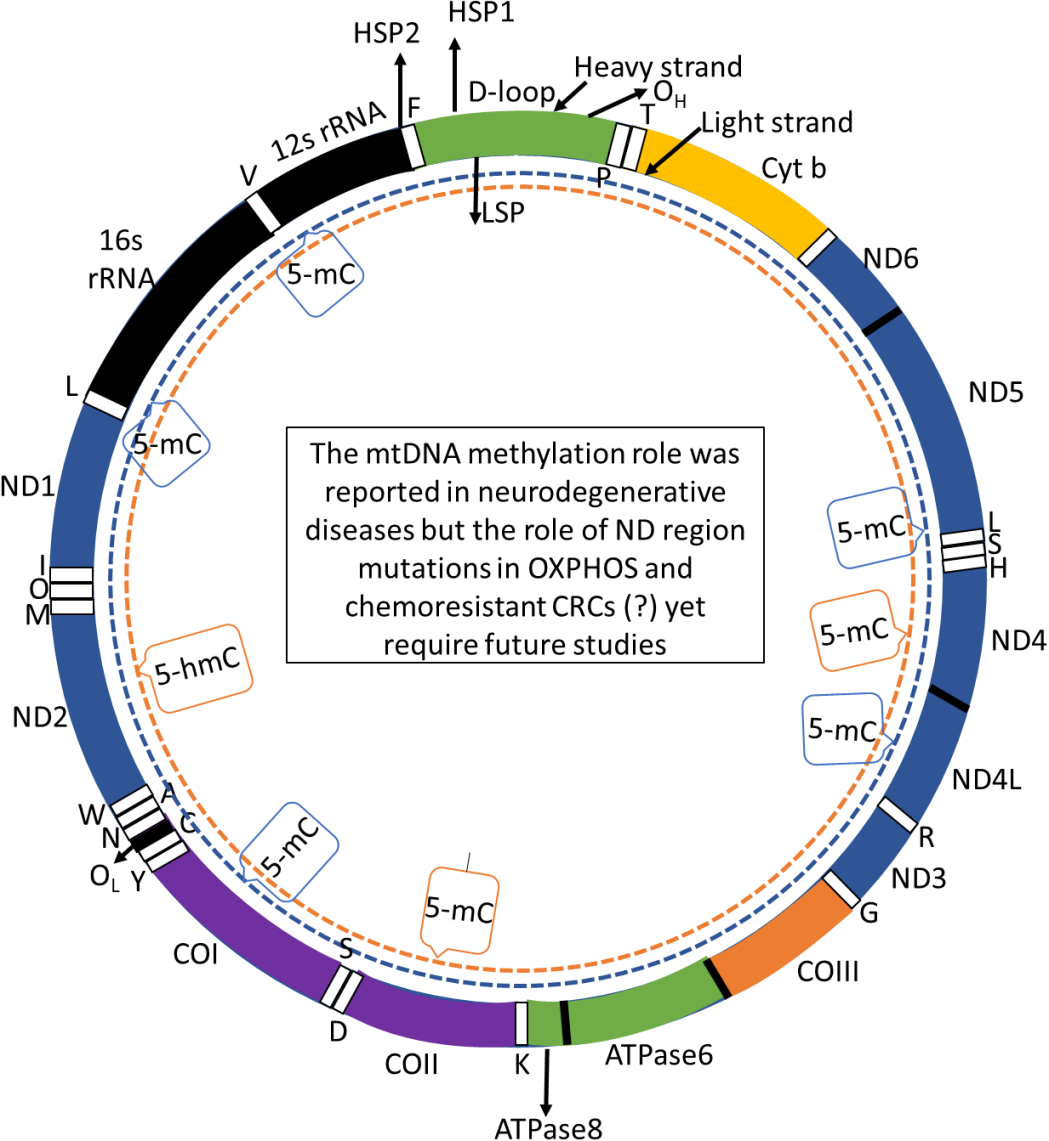
mtDNA genome consists of 16,569 base pairs associated D loop. This genomic structure contains three essential transcription promoters: the heavy strand promoter 1 (HSP1), responsible for transcribing the two ribosomal RNA genes, HSP2, which drives transcription of the remaining genes on the heavy strand, and the LSP, which manages the transcription of the light strand. The mtDNA encodes 13 essential structural genes, 22 transfer RNAs (tRNAs), and two ribosomal RNAs (rRNAs), crucial for mitochondrial function and cellular energy production. These regions are subject to investigation in studies focused on DNA methylation patterns, particularly in the context of neurodegenerative diseases. Notably, research has examined both global and region-specific DNA methylation (5-methylcytosine, 5-mC) and hydroxymethylation (5-hydroxymethylcytosine, 5-hmC) profiles within mtDNA (17). These epigenetic modifications have been assessed in various tissues obtained from patients with neurodegenerative conditions but these mutations role in the ND regions and their methylation yet to be examined for their potential role in OXPHOS and the progression of chemoresistant colorectal cancers. Abbreviations: COI to COIII: complex I to complex IV.

Mitochondrial D-loop/ND genes mutations and colon cancers: Mutations in D-loop can influence mtDNA transcription, leading to mitochondrial dysfunction and potentially contributing to cancer initiation and progression (128). Elevated ROS levels can have deleterious effects, including apoptosis induction and genomic damage, and can alter cellular fates, shifting from apoptosis to necrosis, which in turn influences nuclear DNA mutations, cell division, and tumor growth. Whether mtDNA variations are causative factors or secondary results of the neoplastic process remains an open question. Given the multifactorial nature of cancer and the critical role of mitochondria in ROS production and apoptosis regulation, further exploration into mtDNA D-loop variations in cancer patients is essential (126). D-loop mutations result in a reduced mtDNA copy number or altered mitochondrial gene expression (**Figure 1**), thereby disrupting mitochondrial metabolism and the oxidative phosphorylation pathway. The exact role of these mutations in cancer progression is still under investigation; however, there is a consensus that mtDNA mutations are valuable cancer biomarkers (126, 129–134). Specifically, mutations within mtDNA displacement loop (D-loop) region have been identified in colorectal and gastric cancers (135) (**Figure 1**). This non-coding region has also been implicated in other malignancies, including lung, colon, ovarian, liver, and breast cancers (136–138). The investigation of mtDNA mutations offers a promising avenue for early cancer diagnostics, as these mutations can serve as potential biomarkers (139). Human mtDNA consists of a 16,569-base pair circular DNA encodes 13 polypeptides essential for the OXPHOS system, along with 12S and 16S rRNA and 22 tRNAs crucial for mitochondrial protein synthesis. D-loop, a critical noncoding region, regulates the replication as well as transcription of mtDNA and contains numerous common polymorphisms, especially within its highly variable segments (140). Spanning 1,124 base pairs (nucleotides 16,024 to 576), D-loop includes hypervariable regions HV1 (16,024-16,383) and HV2 (57-372), which serve as promoters for both the heavy (guanine-rich) and light (cytosine-rich) strands of mtDNA. These regions are particularly prone to mutations in various cancers (136).

Akouchekian et al. (126) analyzed mutation rate within the D-loop in CRC by sequencing mitochondrial control region in 40 CRC patient samples (20 females and 20 males) and comparing them with 150 normal control samples (79 females and 71 males). According to study, a high degree of polymorphism in mtDNA among individuals, with CRC patients exhibiting a higher frequency of variations in the D-loop region compared to controls was evident. Thirteen novel polymorphisms, not previously cataloged in the mitochondrial database (Mitomap), were identified, suggesting a potential link between these mtDNA variations and nuclear DNA mutations in CRC (126). Previous research has demonstrated a link between mtDNA mutations and several cancer incidences in human beings (135, 141, 142).

Mitochondria modulate OXPHOS pathway, which comprises complexes I to V. mtDNA possess seven subunits of complex I such as ND1, ND2, ND3, ND4L, ND4, ND5, and ND6; it also composed of a single subunit of complex III viz., cytochrome b (CYTB), as well as three subunits of complex IV viz., cytochrome c oxidase (CO) I, II, and III (118). Complex I is large and mediate proton-pumping NADH oxidoreductase activity, transferring electrons from NADH to ubiquinone (143, 144). The structure of Complex I includes a peripheral arm that extends perpendicularly to the membrane arm. This peripheral arm is divided into two main sections: distal PD module, composed of ND5 and ND4 subunits, and the proximal PP module, which includes the ND2, ND4L, ND6, ND3, and ND1 subunits to facilitate electron transfer pathway. ND1 subunit, confined to the proximal end, acts as a docking site for the Q module. Complex I is essential for the respiratory chain, playing a critical role in maintaining the NAD+/NADH ratio, regulating ROS levels, generating the mitochondrial membrane potential, as well as producing ATP. Malfunctions in Complex I are often linked to various mitochondrial diseases (118, 145–147).

Mutations in ND genes have profound effects on the malignancy of cancer cells, particularly in invasion and metastasis (146). Research has revealed that ROS-generating mtDNA mutations in ND6, such as G13997A and 13885insC cause higher metastasis in cancers (115, 117). This marked the initial discovery of pathogenic mutations associated with ND genes that driving metastasis (115, 117). Subsequent studies have validated these findings; for instance, the ND5 G13289A mutation has been shown to increase ROS production, invasion in human lung cancer cells (148). Similarly, studies in xenograft models, ND3 G10398A mutation has been observed to increase invasion as well as metastasis in human breast cancer cells, while ND6 missense as well as nonsense mutations exhibit similar effects *in vitro* (119, 149). Furthermore, ND6 gene mutations have been linked to lymph node metastasis in lung adenocarcinoma patients (119).

While some research suggests that increased complex I activity might reduce breast cancer metastasis (150), the connection between decreased complex I activity and metastasis remains ambiguous. The study of ND gene mutations is complicated by their random occurrence in cancer cells and the varied impacts on complex I activity. This study (118) aims to predict the pathogenicity of ND gene to explore their correlation to distant metastasis of CRC cancers (118).

A previous study (118), described that nonsynonymous single-nucleotide variants (SNVs) and SNPs in ND genes of NSCLC and colon cancer. Candidates likely to reduce complex I activity were selected based on Grantham value, evolutionary conservation, as well as protein structure and indicated a significant association between these SNVs and SNPs with distant metastasis (118).

ND6 13885insC mutation, in particular, has been shown to foster metastasis in low-metastatic cells. P29mtB82M cells with this mutation exhibit lower complex I activity, higher ROS production, and greater lung-colonizing ability compared to P29mtP29 cells with wild-type mtDNA (115, 118). P29mtB82M cells possess higher spontaneous metastatic potential, forming more metastatic foci. In this scenario, upregulation of metastasis-related genes such as Mmp11, Plaur, Ccl7, c-Myc, K-ras, Cd44, and VEGF-A (118).

The mutation induced a shift towards enhanced aerobic glycolysis (146), upregulating genes encoding for proteins include Glut1, hexokinase 1, phosphoglycerate kinase 1, and phosphofructokinase 1, while suppressing pyruvate dehydrogenase kinase 1. HIF-1a levels were upregulated which contributing to resistance against severe hypoxia. These changes suggest that the ND6 13885insC mutation enhances metastasis by stimulating metastasis-related genes, as well as metabolic reprogramming, tumor angiogenesis (118, 151, 152). Elevated expression of genes related to metastasis include Mmp11, Plaur, Ccl7, Kras, Myc, CD44, and VEGF-A. MMP11 and Plaur play roles in degrading the extracellular matrix (153, 154), while Ccl7 recruits tumor-associated macrophages, enhancing malignancy (155–157). Kras and Myc contribute to increased malignancy (158–160), whereas CD44 denotes a marker for cancer stem cells associated with metastasis (161). VEGF expression was higher in P29mtB82M cells, promoting tumor angiogenesis. Interestingly, upregulation in metastasis suppressor gene Mtss1 was evident whereas other metastasis enhancer genes Pnn, Lpar6, and Fxdy5 exhibited low expression, yet metastasis ability is more. Enhanced glycolysis and downregulation of PDK1 suggested increased acetyl-CoA generation. Increased HIF-1α expression in P29mtB82M cells likely led to upregulation of VEGF-A and glycolytic enzyme genes, contributing to hypoxia resistance and metastasis. These phenotypic changes collectively result in higher distant metastasis in P29mtB82M cells (118, 162).

As we discussed above, mtDNA related mutations occur randomly, leading to variability in each cancer cell. Despite this randomness, pathogenic missense as well as nonsense mutations in ND genes found to be crucial for distant metastasis (115, 117, 118, 149, 150). The study report by Nobuko Koshikawa et al. (118) sequenced genes such as ND1, ND2, ND3, ND4L, ND4, ND5, and ND6 in tissues from 45 primary NSCLC tumors and 37 brain metastases, as well as 22 primary colon cancer tumors and 11 distant metastases. They identified 51 somatic mutations which include a total of 22 nonsynonymous and 29 synonymous type, with a higher mutation frequency in ND6 compared to other ND genes. These mutations appeared as overlapping peaks (heteroplasmy) or single peaks (homoplasmy) on electropherograms, with homoplasmy being less prevalent in metastatic lesions (118).

### 4.1 ND gene mutations and colorectal cancer metastasis

Thus, ND gene mutations are predominantly associated with metastasis experimentally (115, 117, 118, 149, 150). Nobuko Koshikawa et al. (118) described pathogenic SNVs as well as SNPs in ND genes, indicating involvement of complex I deficiency in metastasis and selected 12 SNVs as well as 2 SNPs. Furthermore, according to evolutionary conversation studies, SNPs *T3394C* and SNVs *T3398C, G3709A, T10363C, C11409T, T12338C, G13103A*, and *T14138C* involve conserved amino acid mutations. SNPs T3394C and C3497T and SNV T3398C are linked to mitochondria-related diseases. SNP C3497T and SNVs C3689G, G3709A, and G3955A may cause conformational changes in the ND1 protein, affecting complex I activity (143, 144) (**Table 4**). Complex III transfers electrons and generates ROS (163, 164), so its dysfunction may cause more severe oxidative stress than complex I. Dysfunctions in complexes IV and V mitigate generation of ATP. Complex I dysfunction produces moderate ROS levels, promoting cell proliferation and survival, thereby favoring cancer cell metastasis (165). Homoplasmic states are observed to be minimally prevalent in cancer cells undergoing metastasis, possibly due to the pathogenic nature of heteroplasmic mutations (122, 145). The heteroplasmic state correlates with breast cancer invasion (118).

**Table 4:** ND (NADH dehydrogenase/Complex I) gene mutations gene mutations, metastatic phenotype, mechanisms, and contribution to colorectal cancer metastasis.

Cancer type	ND gene mutation(s)	How it induces/associates with metastasis	How specific mtDNA mutations contribute to CRC metastasis	Refs
Non-small cell lung cancer (NSCLC) & Colon cancer (clinical associations)	Multiple pathogenic SNVs and SNPs across ND genes (12 SNVs + 2 SNPs selected by Koshikawa et al.; examples: T3394C, T3398C, G3709A, T10363C, C11409T, T12338C, G13103A, T14138C, C3497T, C3689G, G3955A)	Complex I deficiency → altered electron transport and moderate ROS production → ROS signaling (sub-lethal) promotes proliferation, survival, and metastatic traits; heteroplasmy may favor invasion. Association reported clinically with distant metastasis in NSCLC and colon cancer, but causality requires functional validation.	Many of these ND variants are postulated to reduce Complex I activity (via conserved aa changes or conformational impacts), producing signaling ROS that can promote metastatic programs (migration, invasion, EMT-like phenotypes) and correlate with distant metastasis in clinical cohorts. Functional confirmation for each variant (activity, ROS output) is needed.	(115, 117, 118, 149, 150) (122, 145)
Colorectal cancer (CRC) - specific ND6 mutation	ND6 13885insC (frameshift/truncating event)	Reprograms cellular energy metabolism, upregulates metastasis-related gene expression, promotes angiogenesis and invasive phenotypes resulting in enhanced metastatic potential in experimental models.	Direct experimental evidence indicates this ND6 insertion increases metastatic behavior of CRC cells by shifting metabolic flux toward supportive pathways for invasion and by inducing pro-angiogenic and pro-invasive transcriptional programs.	(118)
Colorectal (general) - conserved/disease-linked ND variants	T3394C, C3497T, T3398C, G3709A, C3689G, G3955A, T14138C, etc. (conserved aa or disease-linked SNP/SNVs)	Conserved amino-acid altering variants and SNVs can change ND1/ND subunit conformation → reduced Complex I efficiency → altered ROS/bioenergetic signaling that favors metastatic phenotypes (proliferation, survival).	In CRC these conserved ND variants are hypothesized to impair Complex I, generating signaling ROS levels that support tumor cell survival and dissemination; several (e.g., C3497T, C3689G, G3709A, G3955A) may cause conformational changes in ND1 impacting activity. Functional readouts remain necessary.	(143, 144) (118)
Multiple cancer types (mechanistic context)	Heteroplasmic vs homoplasmic ND mutations (heteroplasmy common in metastatic cells)	Heteroplasmy of pathogenic mtDNA variants often correlates with invasive behavior (heteroplasmic mutations provide a dynamic range of mitochondrial dysfunction that can be selected during progression); homoplasmy of strongly pathogenic variants is rare in metastasis due to deleterious effects.	Heteroplasmic ND variants in CRC may permit sub-lethal Complex I dysfunction and adaptive ROS signaling that promote metastasis, whereas fixation (homoplasmy) of strongly deleterious variants is selected against in metastatic clones.	(118, 122, 145)
Colorectal cancer - mtDNA copy number/mito-epigenetics (contextual contribution to metastasis)	(Not ND-gene specific) ↑ mtDNA copy number; TFAM loss/depletion (mtDNA depletion in some contexts)	Increased mtDNA content enhances OXPHOS capacity and supports metastatic phenotypes in MSS CRC; conversely, TFAM-mediated mtDNA depletion can promote progression and chemoresistance in MSI CRC — illustrating that mtDNA quantity and regulation modulate metastatic potential via metabolic reprogramming.	In MSS CRC, elevated mtDNA promotes cell survival, OXPHOS dependence, and metastasis (*in vitro* and *in vivo*). Thus, ND gene mutations act within an mtDNA-quantity and mitoepigenetic landscape that can amplify or modulate their pro-metastatic effects.	(168, 169) (170–175) (30, 176–186)
Broad mechanistic comparison (other complexes)	Complex I ND mutations vs Complex III/IV/V dysfunction	Complex I dysfunction → moderate ROS that promote proliferation/survival (pro-metastatic). Complex III dysfunction → higher ROS and more severe oxidative stress. Complex IV/V defects → ATP reduction and different downstream effects.	ND (Complex I) mutations in CRC are specifically implicated in generating a pro-survival ROS milieu that can drive metastasis, particularly when combined with increased mtDNA/OXPHOS reliance in CRC cells.	(163, 164) (165)

ND gene mutations and, their experimentally observed associations with metastasis (including NSCLC and colorectal cancer), proposed mechanisms, and how specific mtDNA changes contribute to CRC metastasis. aa: amino acid.

By selected SNVs and SNPs across ND genes, Nobuko Koshikawa et al. (118) described a profound interlink with distant metastasis in NSCLC and colon cancer. However, this association is based on presumed pathogenicity and requires confirmation by examining each mutation’s impact on complex I activity as well as the production of ROS. Pathogenic ND gene mutations likely influence metastasis across various cancer types. Complex I subunits are encoded by 44 genes confined to mtDNA and nuclear DNA, with mutations in 21 nuclear genes decreasing complex I activity, potentially affecting metastasis. Establishing a novel experimental system to study the impact of ND gene mutations on metastasis in various cancer cells is essential, aiming for advanced therapies and precise cancer prognosis pf CRCs (117, 119, 148, 149, 166, 167).

In summary, according to these reports, ND gene mutations impact distant metastasis in NSCLC and colon cancer. ND6 13885insC mutation enhances metastasis by reprogramming energy metabolism, upregulating metastasis-related genes, and enhancing tumor angiogenesis (**Table 4**). A previous report identified pathogenic ND gene SNVs and SNPs associated with distant metastasis. Future studies warranted to provide insight into ND gene mutations’ role in cancer metastasis and suggests novel therapeutic targets (118).

## 5 Mitoepigenetics and metastatic colorectal cancer: advancing investigations

Mitochondria, pivotal for cellular metabolites and energy, frequently exhibit varied dysfunctions in cancers, including CRC. Long-established Warburg effect characterizes cancer cells, emphasizing glycolysis and oxidative metabolism dysregulation, yet CRC uniquely relies on mitochondrial OXPHOS as its primary energy source. In addition, extent of mitochondria in CRC tissues surpasses that in normal colon mucosa, underscoring mitochondria’s critical, albeit unclear, role in CRC progression (168, 169).

Mitochondria possess their genome encoding 13 polypeptides crucial for electron transport chain and ATP synthase. Variations in mtDNA copy numbers correlate closely with various cancers: decreased in gastric, breast, hepatocellular, NSCLC, and renal cell carcinoma, yet increased in CRC (170–174). Recent studies implicate mtDNA depletion via TFAM mutation in fostering tumor progression as well as cisplatin resistance in microsatellite instability (MSI) CRC, with implications yet to be fully explored in microsatellite stable CRC (175) (**Table 4**). This study systematically explores how altered mtDNA copy numbers functionally affect MSS CRC progression, demonstrating that increased mtDNA promotes cell survival and metastasis via enhanced mitochondrial OXPHOS, suggesting novel therapeutic targets (30, 176–180).

OXPHOS predominantly generates cellular energy, with mtDNA encoding ETC components crucial for its function (187). CRC studies reveal increased mtDNA copy numbers, notably in early stages, implicating mtDNA in CRC initiation (181–183). A few other reports demonstrating that elevated mtDNA promotes MSS CRC cell survival, proliferation, and metastasis *in vitro* and *in vivo* (184–186). This contrasts with MSI CRC, where lower mtDNA levels correlate with increased glycolysis and chemoresistance (175). Differential mtDNA content may underpin these metabolic differences, necessitating further mechanistic studies (188) (30).

Mitochondria’s modest 13-gene genome contrasts with over 2000 proteins influencing diverse functions, including miRNAs originating from mitochondria (mitomiRs), regulating nuclear mRNA translation and cell phenotype (189–194). Xiacheng Sun et al. (30) described decreased mitochondrial COXIV-1 in CRC adenomas, crucial for mitochondrial-encoded complex IV and V regulation, affecting oxidative phosphorylation and ATP production (195, 196). Notably, miR-210 targets COX10, linking mitomiRs to CRC pathogenesis (197). Understanding these pathways aids in clarifying colorectal adenomatous polyps’ clinicopathological characteristics and early detection strategies (198). For instance, CRC pathogenesis involves mutations in tumor suppressor (e.g., P53, APC) and oncogenes (e.g., KRAS), regulated post-transcriptionally by miRNAs, influencing diverse cancer pathways (197, 199–204). MitomiRs, such as miR-21, miR-210, are implicated in ROS regulation, critical in CRC due to mitochondrial ROS production during oxidative phosphorylation (205–207). Mitochondrial gene expression changes during adenoma-carcinoma progression, with age-related accumulation of dysfunctional mitochondria contributing to CRC pathogenesis (206, 207). Studying mitomiRs (e.g., miR-24, miR-181, miR-210, miR-21, miR-378) across colorectal adenomatous polyps reveals varied expression patterns correlating with tumor architecture and progression, suggesting their regulatory roles in mitochondrial functional pathways (201, 204, 208–211).

In conclusion, mitoepigenetic studies demonstrate mitochondrial dynamics’ pivotal role in CRC evolution from adenomatous polyps to adenocarcinomas, urging further investigation into these intricate pathways for therapeutic and diagnostic advancements.

The impact of demethylation of D-loop region of mtDNA on mtDNA copy number, cell cycle progression, apoptosis, and cell proliferation in CRC remains uncertain (212). For instance, 5-AZA acts by irreversibly inhibiting DNA methyltransferases once incorporated into DNA, a mechanism predominantly utilized in treating hematologic malignancies and potentially applicable to other cancer types, including CRC (213). Numerous studies have indicated that 5-AZA can lead to reduced cell viability and a higher apoptotic rate in different CRC cell lines (214–219). Variations in results across studies might be attributed to differences in incubation periods and 5-AZA concentrations. For instance, Mossman et al. observed cell death in SW480 cells after a 72-hour incubation with 15 µM 5-AZA, whereas HCT116 cells did not exhibit cell death under the same conditions (218). Furthermore, zebularine, a similar DNA methyltransferase inhibitor, was found to stimulate Colo-205 cell growth at concentrations above 45 µM (218). Consequently, relatively minimal concentrations of 5-AZA (up to 10 µM) used for 24 hours in this study which has not induced a strong inhibition on CRC cells (219). Treatment of Colo-205 and Lovo colorectal cancer cells with 5 µM 5-AZA revealed notable alterations in mitochondrial and cell cycle dynamics. In Colo-205 cells, increased cell viability, delayed G0/G1 phase progression, minimal apoptosis, and elevated mitochondrial DNA (mtDNA) copy numbers were observed, while Lovo cells exhibited enhanced proliferation and mtDNA content following similar treatment. These findings suggest that elevated mtDNA levels may drive metabolic adaptation, providing the additional energy required for accelerated proliferation. Consistent with previous studies showing that mtDNA depletion impairs growth in breast and glioblastoma cells (220–223), the current observations imply that mtDNA abundance is closely linked to proliferative capacity. The extended G0/G1 phase in Colo-205 cells likely reflects an increased demand for mtDNA synthesis preceding genomic DNA replication, supporting the hypothesis that mitochondrial biogenesis and replication are tightly coordinated with cell cycle progression. Differences between Colo-205 and Lovo responses further highlight cell line-specific regulatory mechanisms governing mito-nuclear crosstalk during chemotherapeutic stress (220–223).

While mtDNA variation and demethylating agent studies in CRC offer intriguing mechanistic insights, it is critical to frame these findings accurately and avoid over-interpretation. For example, although a large Chinese cohort found that mtDNA haplogroup M7 was associated with worse prognosis in CRC in a northern Chinese population, this remains a population-specific finding and does not establish mtDNA haplogroups as reliable predictive or causative biomarkers across global CRC cohorts (212, 224). Likewise, studies of ND-gene mutations (such as ND6 13885insC) and OXPHOS upregulation in model systems demonstrate potential functional effects but lack evidence in large human metastatic CRC datasets to support the claim that these mutations drive metastasis rather than being passenger or adaptive events. In relation to epigenetic therapy, while 5-azacytidine (5-AZA) has been shown to alter mtDNA D-loop methylation and copy number in certain CRC cell lines (e.g., Colo-205) induced by treatment, this effect is highly cell line-specific and does not yet translate into robust clinical data addressing chemoresistance in CRC (212, 224).

D-loop encompassing 1122 base pairs, is critical for initiating mtDNA transcription and replication (225). While the role of D-loop methylation in mtDNA function is established, its relationship with mtDNA copy number is less understood. Various factors, including TFAM, that interacts with mtDNA and promotes transcription through the formation of initiation complex, and it can influence mtDNA copy number (226). Demethylation of these sites result in the enhanced number of mtDNA copy number, consistent with previous research linking demethylated D-loop regions to higher mtDNA copy numbers in CRC (227). Demethylated CpG sites might enhance mtDNA replication by facilitating TFAM binding and mtDNA transcription initiation, although further research is necessary to confirm this hypothesis (228) (212). Overall, demethylation across specific CpG sites in D-loop promoter may result in a higher mtDNA copy number in CRC, influencing biological behaviors such as enhanced cell proliferation and modulation of cell (212).

In conclusion, the demethylation of specific CpG sites in D-loop promoter may increase the overall copy number of mtDNA in CRC, leading to increased cell proliferation, reduced apoptosis, and a delay in the G0/G1 phase. Thus, DNA methylation influence at D-loop region of mtDNA on the expression of rate-limiting enzymes, but their impact on OXPHOS in CRC remains unclear. Thus, the quantitative changes in ND2 expression as well as methylation across D-loop at the time of CRC progression, along with potential correlations to clinicopathological stages (106). In a study by Shi Feng et al, tumor and noncancerous tissues were subjected to surgical resection from 44 patients diagnosed with CRC. Authors evaluated Cox IV and ND2 expressions in all the specimens obtained from these patients. Correlating these findings with clinicopathological data revealed an association between changes in ND2 expression and clinicopathological stage of CRC (106). The increase in ND2 expression was evident as early as stage I and continued to rise through stages I to IV. Additionally, the proportion of unmethylated D-loop enhanced in tumor as well as non-cancerous tissues, paralleling the rise in ND2 expression (106) (**Figure 1**). Results indicated a higher ND2 expression in tumor tissues than non-cancerous tissues. D-loop region was methylated in 79.5% of non-cancerous tissues, while this percentage dropped to 11.4% in tumor tissues (106). This demethylation likely enhances mitochondrial function, contributing to the metabolic reprogramming observed in cancer cells (106).

Changes in mtDNA copy numbers are recognized as a crucial hallmark of cancers but the quantitative changes in mtDNA should be explored to vividly examine the initiation or progression of CRC (182). Shi Feng et al., 2011 (182) investigated quantitative alterations in mtDNA copy number during CRC initiation and progression and explores potential correlations with clinicopathological stages. Authors in this study surgically resected both tumor tissues as well as noncancerous tissues from 24 colon cancer patients and 20 rectal cancer patients respectively. mtDNA copy numbers were ascertained and the results of this study described the significant raise in mtDNA copy numbers in the CRC tissues when compared to noncancerous tissues (182). Furthermore, correlation with clinicopathological data revealed that changes in mtDNA copy number were associated with clinicopathological stage of CRC, with a marked increase in stages I and II (182). No significant association with gender was observed. Increased mtDNA content could enhance cellular energy production and biosynthetic capacity, supporting rapid cell proliferation and tumor growth. These findings suggest the significance of mtDNA copy number for the initiation as well as progression of CRC especially in the early stages (182).

The role of D-loop hypomethylation in regulating mtDNA copy number as well as ND2 expression in CRC remains unclear (227). This study investigates the association between D-loop methylation status, mtDNA copy number, and ND2 expression in 65 CRC tissue samples and the surrounding non-cancerous tissues. Additionally, a demethylation experiment conducted on Caco-2 CRC cell line using 5-Aza (227). Results of this study (227) described that typical decline in methylation across D loop in CRC tissues when compared to non-cancerous tissues was evident which characterized by the decline in D-loop methylation in clinicopathological stages III and IV than the stages I and II (227). According to this report, the demethylation of D-loop has resulted in the higher mtDNA copy number and ND2 levels. In addition, 5-AZA treatment increased mtDNA copy number as well as ND2 expression in Caco-2 cells (227). Future reports should describe the function of D-loop demethylation in CRC by exploring how D-loop demethylation influences mtDNA replication and ND2 gene expression (227). It is crucial to assess the efficacy of targeting D-loop methylation with demethylating agents or other epigenetic modulators in chemoresistant CRC treatment. By advancing the exploration of the epigenetic regulation of mtDNA in colorectal cancer, these studies could explore the novel epigenetic-based therapeutic strategies to ameliorate CRC or chemoresistant CRC (227).

It is crucial to ascertain the impact of demethylation in D-loop of mtDNA on mtDNA copy number and subsequent effect on the CRC cell proliferation, cell cycle. A previous study by Huan Tong et al. (212) described the higher mtDNA copy number in Colo-205 and Lovo cells upon 5-AZA treatment and the rate of cell cycle and apoptosis is higher upon this treatment. In these cell lines, enhanced methylation was evident at 4^th^ and 6^th^/7^th^ CpG regions of D-loop which was mitigated upon 5-AZA treatment. However, the cell cycle and mtDNA copy number has not changed upon 5-AZA treatment in the CRC cell lines include HCT116, SW480, LS-174T, and HT-29 cells (212). Furthermore, authors have not observed any alterations in demethylation at 4^th^ and 6^th^/7^th^ CpG regions of D-loop in these CRC cell lines upon 5-AZA treatment (212). Further research should explore the molecular mechanisms by which D-loop demethylation influences mtDNA replication and gene expression, including the role of transcription factors like TFAM in this process. It is crucial to explore the interplay between nuclear and mitochondrial epigenetic regulation and its impact on cancer metabolism, progression, and resistance to therapy in CRC (212). For instance, TFAM can regulate both mtDNA transcription and replication. Elevated TFAM expression has been observed in drug-resistant hepatocellular carcinoma cells, and its inhibition has been shown to restore the chemosensitivity of these resistant cells, suggesting a potential therapeutic target for overcoming chemoresistance (229, 230). *In vivo* model of Kras-driven lung cancer, deletion of the TFAM gene results in compromised mitochondrial function, leading to a reduced incidence of tumors (231). In CRC cells, there is an increase in mitochondrial divalent uptake that triggers the activation of phosphodiesterase 2, which in turn inhibits mitochondrial protein kinase A. This inhibition stabilizes the accumulation of TFAM within the mitochondria, fostering cell proliferation (232). Similarly, enhanced mitochondrial calcium uptake has been linked to upregulated TFAM expression, which fostering mitochondrial biogenesis and increases the production of mitochondrial ROS. This cascade activates the NF-κB signaling, thereby accelerating the progression of CRC (233).

**Figure 2:**
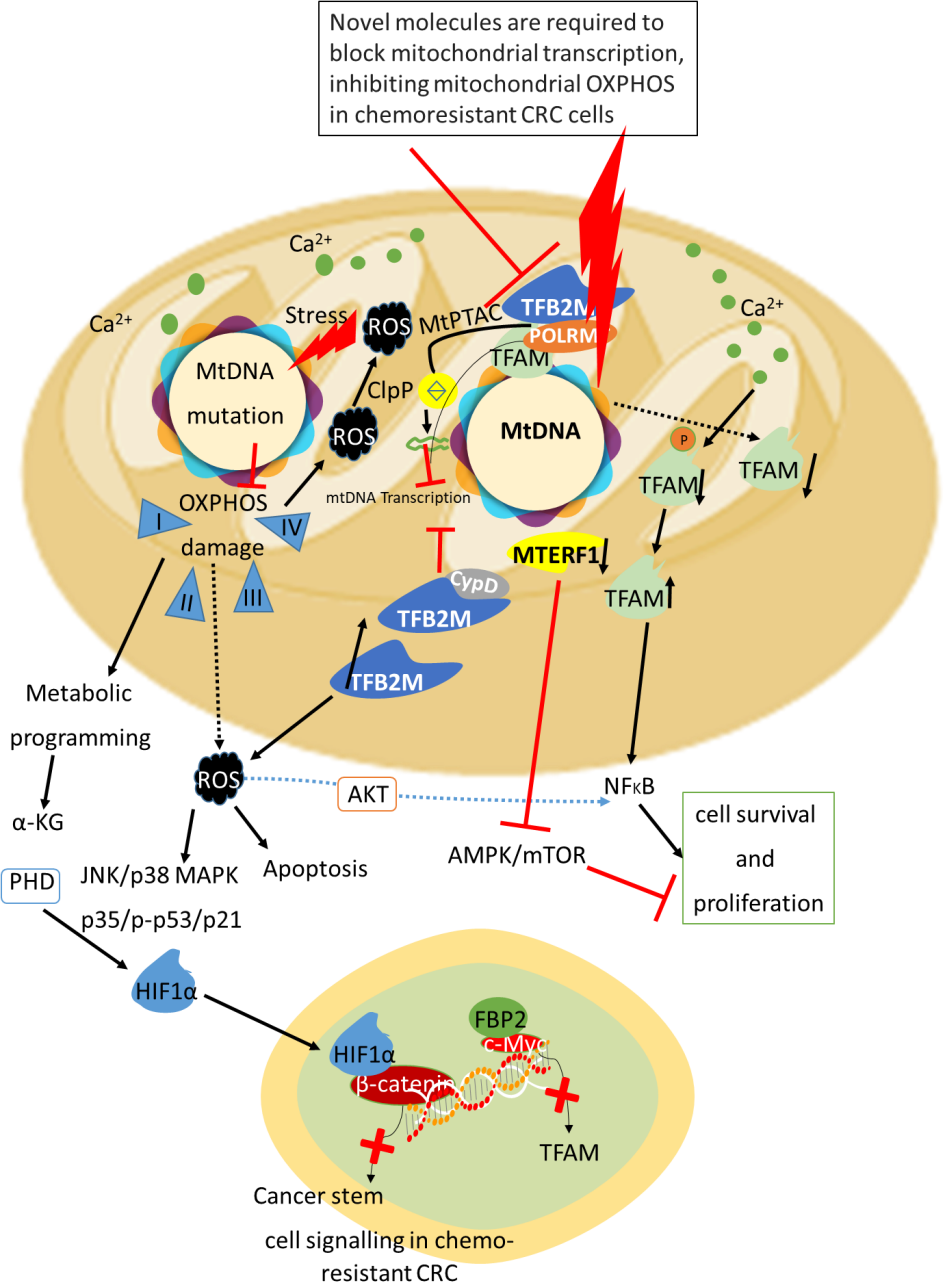
Impact of mitochondrial DNA mutations and transcriptional inhibition on oxidative phosphorylation, CRC tumor cell dynamics, and epigenetic regulation. Mitochondrial DNA (mtDNA) mutations, alongside the impairment of mitochondrial transcription, lead to impaired OXPHOS, which consequently elevates reactive oxygen species (ROS) production. This ROS surge modulates critical signaling pathways, including MAPK/mTOR, other cell survival pathways like Akt thus influencing the proliferation of cancer cells. In this context, the silencing of mitochondrial transcription factor A (TFAM) triggers metabolic reprogramming within tumor cells, resulting in the release of alpha-ketoglutarate (α-KG). This metabolic shift can cause downregulation in β-catenin, thereby block stem cell signaling and attenuating oncogenic potential (229). Inhibition of TFAM expression promotes mtDNA release into the cytoplasm, activating oncogenic signaling and highlighting the potential of targeting mitochondrial RNA polymerase (POLRMT) to suppress OXPHOS and tumor proliferation in chemoresistant metastatic CRC. Furthermore, variability in mtDNA mutations and D-loop methylation across cancers elucidates the need to explore epigenetic modulation of mtDNA as a therapeutic strategy to overcome chemoresistance and improve colorectal cancer treatment outcomes.

Mutations in TFAM have been implicated in promoting increased cell proliferation and enhanced tumorigenic potential in xenograft models. Notably, silencing TFAM in CRC cells induces metabolic reprogramming (229, 230). This silencing disrupts the Wnt/β-catenin signaling pathway via an increase in α-ketoglutarate levels, ultimately inhibiting tumor initiation and progression (234, 235). MTERFs (mitochondrial transcription termination factors) comprise a protein family that, despite sharing structural homology, perform diverse functions crucial to mitochondrial homeostasis. Disruption of MTERF activity has been shown to impair mitochondrial function, leading to mitochondrial damage and contributing to the pathogenesis of various mitochondrial-related diseases (236–238). The precise relationship between MTERF proteins and processes such as OXPHOS, cell proliferation, and tumorigenesis remains incompletely demonstrated in proliferating CRC cells. However, research has indicated that MTERF1 can modulate mitochondrial gene expression and OXPHOS levels. In HeLa cells, overexpression of MTERF1 has been shown to enhance mitochondrial gene transcription, increase OXPHOS activity, and elevate cyclin D1 levels, which promotes cell proliferation. In contrast, MTERF1 knockdown leads to diminished ATP generation, lower cyclin D1 expression, as well as cell cycle arrest (239). Upregulated expression of MTERF1 in CRC cells could cause higher cell division and enhance migration and invasion of cancer cells to form tumors. Mechanistically, MTERF1 could control AMPK/mTOR pathway, which influences mtDNA replication, transcription, subsequently modulate protein synthesis (229). Additionally, MTERF1 overexpression reduces ROS production, further enhancing mitochondrial activity for OXPHOS and contributing to cancer progression (229, 240) (**Figure 2**). Moreover, the inhibition of TFAM expression facilitates the release of mtDNA into cytoplasm, and activates signaling pathways to modulate oncogenesis and it is crucial to develop novel small-molecule compounds to target mitochondrial RNA polymerase (POLRMT) to block mitochondrial transcription, thereby selectively blocking OXPHOS and curbing tumor cell proliferation in chemoresistant metastatic CRCs. These findings prompt questions regarding the role of oncogenes in tumorigenesis in terms of association with mtDNA mutations or SNPs and whether the combined effects of mtDNA as well as nuclear DNA quantitative trait loci might explain the variability in cancer susceptibility and metastatic potential across different individuals. mtDNA mutations exhibit variability in both frequency and location among various cancers, with prostate, stomach, and colorectal cancers showing the greater mutation rates. Given this variability, it is crucial to investigate the potential of targeting D-loop methylation through the use of demethylating agents or other epigenetic modulators as a therapeutic strategy, particularly in chemoresistant colorectal cancer. By deepening our understanding of the epigenetic regulation of mtDNA in CRC, these studies pave the way for the development of novel epigenetic-based therapies to combat CRC, including its chemoresistant forms.

MTERF3 has been recognized as an oncogene across multiple cancer types, with gene amplification and elevated expression of MTERF3 levels strongly correlating with poor overall survival rates in cancer patients (241). Its overexpression has been shown to promote tumorigenesis both *in vitro* and *in vivo*, and enhancing proportion of cells in S phase of the cell cycle, thereby accelerating proliferation (242). In CRC specifically, MTERF3 has been implicated in the upregulation of pro-inflammatory cytokines such as IL-6 as well as IL-11, that not only modulate tumor growth but also enhance resistance to radiotherapy (243). These findings described the oncogenic role of MTERF3 in promoting both tumor progression and therapeutic resistance (229). However, in-depth exploration is crucial to uncover the intricate molecular signaling by which TFAM and MTERF family members regulate mitochondrial function and influence metastasis and chemoresistance. Specifically, studies should focus on understanding how these proteins modulate mitochondrial biogenesis, energy production, and ROS signaling in different chemoresistant cancers including CRC (229). Given their pivotal role in mitochondrial regulation and cancer progression, TFAM and MTERFs hold promise as therapeutic targets. Preclinical studies exploring inhibitors or modulators of these proteins may yield novel treatment options for chemoresistant CRC (229). Furthermore, combining mitochondrial-targeted therapies with conventional cancer treatments like chemotherapy or radiotherapy could improve efficacy and overcome resistance. For example, TFAM or MTERF inhibitors could be used alongside traditional treatments to synergistically halt tumor growth and sensitize cells to therapeutic interventions. Future studies must focus on deciphering how mtDNA single nucleotide polymorphisms and interactions with nuclear DNA quantitative trait loci influence cancer susceptibility and metastatic behavior. Furthermore, targeting epigenetic modifications within the mitochondrial genome especially through D-loop demethylation or the application of epigenetic modulators represents a promising therapeutic strategy to reprogram mitochondrial function and overcome chemoresistance. Collectively, these insights reinforce the emerging paradigm that mitochondrial epigenetic regulation serves as a pivotal determinant of CRC progression and therapeutic response, providing a conceptual framework for developing next-generation mitochondrial-targeted and epigenetic-based therapies.

## 6 Integrative immunotherapeutic and metabolic strategies to overcome chemoresistance in metastatic colorectal cancer

Immunotherapy has emerged as a transformative approach in mCRC, yet its clinical efficacy remains largely confined to a limited subset of microsatellite instability-high (MSI-H) tumors. The majority of microsatellite-stable (MSS) mCRC cases continue to rely on combinations of chemotherapy with targeted pharmacotherapies such as anti-VEGF (e.g., bevacizumab) and anti-EGFR (e.g., cetuximab, panitumumab) drugs (33). Despite their established therapeutic benefit, both modalities are hampered by intrinsic and acquired resistance mechanisms that arise from tumor heterogeneity, dynamic clonal evolution, and metabolic adaptation.

Anti-VEGF therapy, a standard-of-care since 2004, effectively suppresses angiogenesis but lacks well-defined molecular predictors of responsiveness. Conversely, anti-EGFR therapy provides benefit only in RAS and BRAF wild-type patients, excluding approximately 60% of CRC cases due to mutation-driven intrinsic resistance (33, 244). Even in responsive subgroups, selective pressure under anti-EGFR therapy rapidly fosters secondary mutations within the EGFR extracellular domain and downstream MAPK pathway components, conferring adaptive resistance (33, 244). Longitudinal circulating tumor DNA (ctDNA) profiling now enables noninvasive detection of these emergent mutations, uncovering a dynamic interplay between drug exposure, clonal selection, and treatment relapse. Moreover, 8q chromosomal gains, frequently encompassing MYC amplification, have been correlated with resistance to EGFR blockade, supporting the rationale for combined EGFR and pan-KRAS inhibition as a next-generation therapeutic strategy (33, 244) (**Table 5**).

**Table 5:** Integrated overview of molecular modulators, metabolic and immunotherapeutic strategies to overcome chemoresistance in metastatic colorectal cancer (mCRC).

Protein/Gene	Modulation in signaling pertinent to CRC	Metabolic strategy to overcome chemoresistance	Novel immunotherapeutic strategy to overcome metastatic chemoresistant CRC	Refs
PIK3CA, APC, MAP2K4, SMAD4, STK11, MAP3K1	Mutation-driven activation of PI3K/MAPK/TGF-β pathways promoting proliferation and drug resistance	Targeting mitochondrial OXPHOS and metabolic rewiring to reduce clonal adaptation	Combined EGFR and pan-KRAS inhibition to overcome adaptive signaling resistance	(32)
KRAS/NRAS/BRAF	Constitutive MAPK activation conferring intrinsic resistance to anti-EGFR therapy	MEK inhibitor (trametinib) plus 5-FU enhances sensitivity by suppressing MAPK pathway	Dual EGFR + KRAS inhibition or adaptive immunotherapy combinations to delay resistance	(33)
EGFR/MYC (8q gain)	Ligand-driven MAPK activation; MYC amplification enhances survival and resistance	Modulating EGFR–MYC signaling axis through metabolic stress induction	Development of EGFR-ligand blockers and anti-EGFR/pan-KRAS dual targeting	(33)
TP53RK	Regulates DNA replication fidelity and cell-cycle checkpoint signaling	CDC7 inhibitor (XL413) induces replication stress and apoptosis	Exploitation of synthetic lethal interactions with DNA repair–modulating immunotherapies	(244)
Drp1/PINK1/Parkin	Mitochondrial fission–fusion control and mitophagy promoting chemoresistance	Inhibition of mitophagy to trigger mitochondrial apoptosis and re-sensitize tumor cells	Integration with immune checkpoint blockade or metabolic adjuvants to enhance T-cell killing	(27)
SLC7A11	Ferroptosis regulator maintaining redox balance and promoting drug resistance	Erastin-mediated ferroptosis induction increases oxaliplatin sensitivity	Combination with immune checkpoint inhibitors to enhance immunogenic cell death	(27)
Cabozantinib (c-MET)/Durvalumab (PD-L1)	Anti-angiogenic and immune-modulatory effects reshape TME	Metabolic normalization and immune microenvironment restoration	Dual inhibition enhances immune infiltration and T-cell activation in MSS CRC	(27)
Nivolumab + Regorafenib	Enhances IFN-γ pathway and immune infiltration	Modulates tumor metabolism and vascular remodeling	Restores cytotoxic T-cell function, particularly in non-liver metastasis CRC	(27)
RNF4/PDHA1	RNF4-mediated ubiquitination degrades PDHA1, promoting glycolysis and metastasis	PDHA1 stabilization reverses Warburg metabolism and limits tumor growth	Combination with metabolic checkpoint inhibitors enhances immune reactivation	(246)
SALL1 (cg13755795 methylation)	Aberrant promoter methylation drives metastatic gene silencing	Demethylation or epigenetic reprogramming to restore tumor suppressive transcription	Integration with epigenetic adjuvants to enhance immune recognition	(245)
RNF43/RNF114	Ubiquitin ligases altering Wnt and NF-κB pathways in CRC	Metabolic suppression through targeted inhibition of RNF114-mediated oncogenic signaling	Use of immunomodulatory agents that target ubiquitin signaling pathways	(36)
Wnt5a/HIF2/EREG	Hypoxia-induced fibroblast signaling sustaining tumor progression	Targeting hypoxia and fibroblast activation to restore angiogenic balance	Blockade of Wnt5a-InfFib axis to enhance immune accessibility and CAR-T efficacy	(37)
METTL3/YTHDC1/NRXN3	m6A-dependent mRNA stabilization regulating metastatic adaptation	Targeting m6A writers/readers to block metastasis-related transcripts	m6A epigenetic therapy integration with immune checkpoint inhibitors	(38)
GNG2	Suppresses metastasis signaling cascades, especially to the brain	Enhancing mitochondrial homeostasis and reducing metastatic migration	Potential biomarker for anti-metastatic immunotherapy	(247)
FSTL3/HIF1α	FSTL3 overexpression stabilizes HIF1α to promote angiogenesis and metastasis	HIF1α inhibitor (KC7F2) reverses FSTL3 oncogenic effects	Combining HIF1α blockade with immune activation to restore anti-tumor immunity	(248)
MitoNIDs (Mitochondria-targeted nanoinducers)	Disrupt mitochondrial metabolism and redox balance	Induce selective mitochondrial dysfunction in tumor cells	Potentiate CAR-T, CD8^+^ T cells, and mRNA vaccine efficacy in MSS CRC	(27)

Expanding beyond receptor-targeted resistance, novel studies have implicated TP53RK as a critical regulator of replication stress tolerance. Its overexpression sensitizes CRC cells to CDC7 inhibition, suggesting a synthetic-lethal vulnerability exploitable through replication checkpoint modulation (244). Similarly, mitochondrial dynamics have been recognized as central to therapeutic resistance, wherein the PINK1–Parkin–Drp1 axis preserves mitochondrial integrity under chemotherapeutic stress. Hyperactivation of Drp1-mediated fission promotes mitophagy and survival, while its inhibition re-sensitizes CRC cells to apoptosis, positioning mitochondrial quality control as a viable metabolic checkpoint for combination therapy (244).

Recent preclinical and translational advances have further illuminated the therapeutic promise of integrating targeted inhibitors, metabolic modulators, and immunotherapies. The MEK inhibitor trametinib, when combined with 5-fluorouracil, demonstrates synergistic suppression of MAPK signaling and enhanced neoadjuvant chemoradiotherapy response. Dual blockade strategies such as cabozantinib (anti-c-MET) plus durvalumab (anti-PD-L1) have shown activity in MSS CRC by concurrently suppressing angiogenesis and reprogramming the tumor immune microenvironment. Moreover, nivolumab–regorafenib co-administration augments T-cell infiltration, particularly benefiting patients without hepatic metastases.

Emerging ferroptosis-based interventions are also redefining metabolic-immune synergy. The SLC7A11 inhibitor erastin enhances oxaliplatin efficacy through iron-dependent oxidative stress, reversing chemoresistance in preclinical CRC models (27). Complementary innovations in nanotechnology have enabled mitochondrial-targeted nanoinducers (mitoNIDs) that disrupt redox balance and potentiate the activity of CAR-T and CD8^+^ T cells, offering new hope for immunotherapy-refractory MSS CRC (27).

At the epigenetic interface, SALL1 promoter methylation and cg13755795 site hypermethylation have been identified as prognostic biomarkers distinguishing CRC from its metastatic counterpart, highlighting the intertwined influence of mitochondrial metabolism and epigenetic regulation in immune modulation (245). Furthermore, RNF4-mediated ubiquitination of PDHA1, a key enzyme bridging glycolysis and the TCA cycle, drives metabolic reprogramming, proliferation, and metastasis linking proteostasis to energy metabolism and chemoresistance (246) (**Table 5**).

Additional molecular mediators, including GNG2, a tumor suppressor limiting brain metastasis and FSTL3, which promotes tumor progression via HIF1α-dependent pathways, have emerged as actionable biomarkers for metastatic control (247, 248) (**Table 5**). Pharmacological inhibition of HIF1α using KC7F2 effectively suppresses FSTL3-driven metastasis, restoring tissue integrity in xenograft and tail-vein models. Collectively, these discoveries delineate an evolving paradigm in mCRC therapy as one that integrates immunomodulation, metabolic disruption, mitochondrial targeting, and epigenetic reprogramming. Such multimodal strategies transcend traditional cytotoxic approaches by simultaneously dismantling tumor survival networks, curbing adaptive resistance, and reactivating antitumor immunity. The convergence of multi-omic profiling, ctDNA-based precision monitoring, and nanotechnology-driven delivery systems is thus redefining the next frontier of precision immunoepigenetic therapy for chemoresistant metastatic colorectal cancer.

### 6.1 Epigenetic regulation of immune checkpoints and TME in colorectal cancer

Epigenetic mechanisms play a foundational role in shaping the tumor immune microenvironment (TIME) in CRC, influencing both tumor-intrinsic and immune-cell–specific transcriptional programs (249). Aberrations in DNA methylation, histone modifications, and chromatin remodeling affect antigen presentation, immune checkpoint expression, and interferon signaling, thereby impacting tumour immunogenicity and immune-evasion capacity (249). For example, hyper-methylation or histone deacetylation of promoters linked to PD-L1, CTLA-4 or T-cell exhaustion genes have been demonstrated in models of CRC, facilitating immune escape (249). Moreover, microsatellite-stable (MSS) CRCs, which typically show poor immunotherapy response, frequently display epigenetic silencing of chemokine genes and reduced effector T-cell infiltration helping to explain the “cold tumour” microenvironment (249).

Recent advances reveal that epigenetic therapies can reprogram the immune landscape of CRC, sensitizing tumors to immunotherapy. DNA methyltransferase inhibitors (DNMTis) and histone deacetylase inhibitors (HDACis) (250) have been shown to restore expression of the antigen presentation machinery, activate endogenous retroviruses, and evoke type I interferon responses collectively converting “cold” MSS tumors into more immunogenic phenotypes (250). In parallel, activation of the cGAS–STING pathway, a key mediator of innate immune sensing has been shown to be epigenetically suppressed in CRC, and re-activation of this pathway through demethylating agents enhances cytotoxic T-cell infiltration and limits metastasis. Collectively, these data support a paradigm in which epigenetic modulation of immune signaling can overcome intrinsic resistance in CRC (250).

Emerging evidence further indicates that epigenetic remodeling directly governs the expression and functional state of immune checkpoint pathways in both tumour and immune cells (251). For example, in CRC, tumour-infiltrating lymphocytes (TILs) display altered DNA methylation and histone-modification profiles at loci encoding PD-1, TIM-3 and TOX2, thereby promoting T-cell exhaustion and reducing checkpoint-blockade responsiveness (251). Moreover, epigenetic reprogramming of cancer stem-like cells (CSCs) enhances immune resistance through metabolic-epigenetic crosstalk that stabilizes PD-L1 expression and suppresses antigen presentation. Understanding how tumour epigenome dynamics and immune exhaustion co-evolve is thus critical to targeting CRC therapy resistance (251). From a translational perspective, integrating epigenetic and immunologic paradigms offers a compelling framework for developing next-generation CRC therapies (251, 252). Combined use of epigenetic modulators with immune-checkpoint inhibitors offers a rational strategy to overcome immune resistance in MSS CRC, which comprises the majority of clinical cases. Furthermore, epigenetic biomarkers such as chemokine-gene methylation signatures or histone-modification profiles hold promise as predictive indicators of immunotherapy response (251, 252). Given that current checkpoint-inhibitor therapies are principally effective in MSI-high CRC, while MSS cases remain refractory, we propose that epigenetic immune-priming strategies are urgently needed to bridge this gap. Future clinical trials in CRC should integrate multi-omic immune-epigenetic profiling to uncover actionable pathways for personalized therapeutic development (251, 252).

### 6.2 Epigenetic regulation of PD-1 blockade response, TME reprogramming, and immunotherapy biomarkers in MSS colorectal cancer

#### 6.2.1 PD-1 blockade in MSS CRC, current efficacy and mechanisms of resistance

Single-agent PD-1/PD-L1 blockade has limited activity in microsatellite-stable (MSS/pMMR) colorectal cancer because these tumors are typically immune-cold, possess lower neoantigen burdens and show active exclusion or suppression of effector T cells (253–255). Recent meta-analyses and systematic reviews confirm negligible objective response rates for ICI monotherapy in MSS CRC, while combination regimens (e.g., PD-1 inhibitors with chemotherapy, anti-angiogenics, or targeted agents) can produce clinically meaningful responses in carefully selected contexts such as neoadjuvant trials or when partnered with immune-modulating partners (e.g., anti-VEGF, MEK inhibitors, IDO1 inhibitors) (253–255). Mechanistically, resistance reflects low baseline antigenicity, WNT/β-catenin and TGF-β–driven T-cell exclusion, suppressive myeloid populations, and epigenetic silencing of chemokine and antigen-presentation genes, all of which blunt PD-1 blockade efficacy in MSS disease (253–255).

#### 6.2.2 TME composition in chemoresistant MSS CRC and epigenetic contributions

Chemoresistant MSS CRCs characteristically exhibit a restructured TME dominated by suppressive myeloid cells (tumour-associated macrophages and MDSCs), regulatory T cells, dysfunctional dendritic cell subsets, and an activated stromal compartment including carcinoma-associated fibroblasts that reinforce immune exclusion (249, 250, 256). Single-cell and spatial transcriptomic studies summarized that epigenetic modifications within both tumour and immune compartments (DNA methylation, histone marks) silence chemokine expression (e.g., CCL5), downregulate antigen-presentation machinery and fix T-cell exhaustion programs thereby stabilizing an immune-cold, chemoresistant phenotype (249, 250, 256). Importantly, these epigenetic programs are dynamic under therapy: chemotherapy can transiently increase neoantigen release but simultaneously select for subclones with epigenetic adaptations that sustain immune evasion. These insights provide a mechanistic link between chemoresistance, immune exclusion, and reversible epigenetic states amenable to therapeutic targeting (249, 250, 256).

#### 6.2.3 Biomarkers beyond MSI/dMMR and harnessing novel immunotherapies via epigenome and tumour heterogeneity

To move beyond MSI/dMMR as the dominant predictive marker, multiparameter biomarker panels are needed that integrate tumour mutational burden/neoantigen quality, spatial immune phenotypes (T-cell inflamed vs immune-desert archetypes), WNT/TGF-β pathway activation, and epigenetic signatures (chemokine promoter methylation, histone modification profiles and chromatin accessibility in tumour and immune cells) (250, 255, 256). Early clinical strategies exploit this biology: epigenetic priming (DNMTi/HDACi or novel chromatin modulators) can upregulate antigen presentation, reactivate endogenous retroviral elements to induce a type-I interferon response, and restore chemokine expression for T-cell recruitment thereby converting MSS tumors into immunotherapy-sensitive states (250, 255, 256). Rational combinations (epigenetic drugs + PD-1 blockade; STING agonists or TGF-β pathway inhibitors + ICIs; oncolytic virotherapy or microbial modulation + ICI) are now in early clinical testing and informed by spatial and single-cell profiling to account for intratumor heterogeneity. Ultimately, integrating epigenomic profiling into clinical trials will enable adaptive selection of combinations tailored to tumour architecture and evolutionary state, offering a clear path to harness novel immunotherapies for MSS, chemoresistant CRC (250, 255, 256).

#### 6.2.4 Mitochondrial signaling to immune modulation and checkpoint therapy resistance

Mitochondrial signaling exerts a central influence on anti-tumor immunity and can directly modulate resistance to immune checkpoint blockade. Mitochondrial metabolism and dynamics regulate T-cell activation, differentiation, and persistence, PD-1 signaling suppresses mitochondrial biogenesis and fission-fusion programs (via PGC-1α and DRP1 pathways), driving T-cell exhaustion and reducing responsiveness to PD-1/PD-L1 inhibitors (257). Cancer-cell mitochondrial dysfunction (e.g., altered OXPHOS, elevated mtROS, mtDNA release, or mitochondrial transfer to immune cells) reshapes the tumor microenvironment toward immunosuppression by promoting regulatory myeloid phenotypes, impairing antigen presentation, and blunting CD8^+^ effector functions include mechanisms that have been linked to poorer immune checkpoint inhibitor (ICI) outcomes (258). Emerging clinical and mechanistic studies even associate host mitochondrial features (haplogroups, mtDNA content) and tumor-derived mitochondrial signaling with differential ICI efficacy, suggesting mitochondria-linked biomarkers of resistance (259). Therapeutically, restoring mitochondrial fitness in exhausted T cells (via metabolic reprogramming, enhancing mitobiogenesis/PGC-1α, or reducing deleterious mtROS) and targeting tumor mitochondrial adaptations are promising strategies to overcome checkpoint resistance and reinvigorate anti-tumor immunity (260). In **CRC**, mitochondrial signaling profoundly shapes immune evasion and resistance to checkpoint blockade therapy. CRC cells frequently display dysregulated OXPHOS and elevated mitochondrial ROS, which promote the secretion of immunosuppressive cytokines (e.g., TGF-β, IL-10) and the polarization of tumor-associated macrophages toward an M2-like phenotype, thereby weakening cytotoxic CD8^+^ T-cell infiltration and activity (27, 261, 262). Additionally, damaged mitochondria in CRC cells can release mtDNA into the cytosol, activating the **cGAS-STING** pathway in a context-dependent manner either enhancing type I interferon mediated immune surveillance or, when chronically activated, promoting immune tolerance and checkpoint therapy resistance (27, 261, 262). Moreover, mitochondrial metabolic reprogramming driven by oncogenic KRAS or p53 mutations enhances OXPHOS reliance in microsatellite-stable (MSS) CRC, which correlates with diminished response to PD-1/PD-L1 inhibitors and a “cold” tumor immune microenvironment. Recent evidence suggests that therapeutic restoration of mitochondrial homeostasis may sensitize CRC to immune checkpoint inhibitors (27, 261, 262). Strategies such as PGC-1α activation, mitochondrial-targeted antioxidants, or OXPHOS inhibitors can recalibrate tumor metabolism and reinvigorate anti-tumor immunity. By modulating mitochondrial biogenesis and redox balance, these interventions reduce ROS-induced immunosuppression, restore T-cell effector functions, and enhance antigen presentation within the tumor microenvironment. Integrating mitochondrial metabolic modulators with immune checkpoint therapy may thus represent a precision immuno-oncology approach for CRC, bridging mitochondrial bioenergetics with durable immune responsiveness (27, 261, 262).

## 7 Clinical epigenetics in metastatic CRC therapies

Epigenetic modifications mediated by histone writer enzymes orchestrate dynamic histone modifications crucial for establishing and maintaining epigenetic landscape (263). These modifications are controlled by a complex interplay among writer enzymes, reader proteins, and erasers. Dysregulation of these processes can drive pathogenesis, including in chemoresistant CRC (263) (**Tables 6**, **7**).

**Table 6:** Selected small molecule inhibitors and PROTACs targeting epigenetic writer proteins.

Protein/Complex	Inhibitor/PROTAC	Reference
EP300/CBP	C646, A485, CBP30, I-CBP112, GNE-781	(267, 269)
PCAF	GSK4037, L-Moses, GSK983/GSK699 (PROTAC)	(273, 274)
EZH2	GSK126, EPZ6438, Tazverik (FDA-approved)	(277, 278)
PRMT5	EPZ015666, HLCL-61, PRMT4 inhibitors	(280, 286)
DOT1L	EPZ5676 (Pinometostat)	(275, 287)

This table summarizes key small-molecule inhibitors and PROTACs targeting epigenetic writer proteins (263).

**Table 7:** Overview of targeted therapies in the various phases of clinical trials (www.cancer.gov/research/participate/clinical-trials) for metastatic colorectal cancer.

Drug	Target	Indication	Combination therapy	Key findings	Refs
Encorafenib (Braftovi)	BRAF protein	Metastatic colorectal cancer with BRAF mutation	Cetuximab (Erbitux)	Significant efficacy in combination with cetuximab	NCI Clinical Trials
Vemurafenib (Zelboraf)	Mutant B-Raf	Colorectal cancer with BRAF mutation	Cetuximab and Irinotecan (Camptosar)	Effective in trials with combination therapy	NCI Clinical Trials
Vitamin D3 (SOLARIS)	N/A	Metastatic colorectal cancer	Chemotherapy and Bevacizumab	Ongoing trial testing the efficacy of adding vitamin D3	NCI Clinical Trials
Tucatinib (Tukysa)	HER2 protein	Advanced colorectal cancer with HER2 overexpression	Trastuzumab (Herceptin)	Tumors shrank or disappeared in over one-third of participants; stable disease in another third	FDA approval documentation, NCI Clinical Trials

Dynamic histone modifications and transcriptional regulation: Histone acetylation, initially linked with transcriptional activation, neutralizes the positive charge on histone tails, thereby modulating DNA-histone interactions and facilitating transcription factor binding and RNA polymerase activity (264). Notably, histone acetylation at H3K27 (H3K27ac) could be considered as the main process at active promoter and enhancer regions, regulating specific gene expression (265). Targeting histone acetyltransferases (HATs) like EP300 and CREBBP, which catalyze H3K27ac, has emerged as a therapeutic strategy to modulate transcriptional programs implicated in CRC progression (266, 267).

Therapeutic targeting of EP300/CBP: EP300 and CBP not only catalyze H3K27ac but also acetylate other histone and non-histone substrates, influencing proliferation and differentiation (268). Small-molecule inhibitors like C646 and A485 selectively target the catalytic activity or bromodomain function of EP300/CBP, respectively, offering potential therapeutic approaches (267, 269). Strategies that simultaneously target multiple domains of EP300/CBP have shown promise in inhibiting CRC cell proliferation synergistically (270, 271).

Exploring selective inhibition and functional roles: Despite structural homology, EP300 and CBP exhibit distinct functional roles in gene regulation and cancer pathogenesis, concluding the need for the development of selective inhibitors (271). Ongoing efforts focus on dissecting their specific contributions to CRC biology using chemical and genetic tools (272).

Expanding Targets Beyond EP300/CBP: Beyond EP300 and CBP, PCAF and GCN5 have emerged as context-dependent regulators in cancer, with inhibitors such as GSK4037 and L-Moses revealing their potential as therapeutic targets (273, 274). Therefore, development of PROTACs like GSK983/GSK699 for PCAF underscores new strategies to modulate protein function and understand their roles in cancer progression and chemoresistance (263).

Histone methylation dynamics: Histone methylation, traditionally considered irreversible, modulates gene expression through diverse mechanisms, including recruitment of TFs and chromatin remodeling complexes (263). Inhibition of histone lysine methyltransferases (PKMTs) like DOT1L and EZH2 has shown therapeutic promise in leukemia and solid tumors, respectively (275, 276).

Targeting EZH2: EZH2, a core component of the PRC2 complex catalyzing H3K27me3, regulates gene silencing in cancer. Inhibitors such as GSK126 and EPZ6438 have demonstrated efficacy in various malignancies, including CRC and glioma (277, 278). Combination therapies involving EZH2 inhibitors with other epigenetic or signaling pathway inhibitors offer synergistic effects against chemoresistance (279) but their efficacy should be examined against chemoresistant/radioresistant CRC.

PRMTs as emerging targets: Protein arginine methyltransferases (PRMTs), implicated in cancer stemness and DNA damage response, are promising targets for therapeutic intervention with inhibitors like EPZ015666 and HLCL-61 showing efficacy in lymphomas and AML (263, 280). Future directions in targeting epigenetic modifiers in chemoresistant CRC include developing selective inhibitors against less explored histone modifications (e.g., serotonylation, crotonylation) and employing innovative strategies like PROTACs to degrade oncogenic proteins (281, 282). Integrating genomic and epigenomic data will identify critical vulnerabilities in CRC, paving the way for personalized therapeutic strategies targeting epigenetic dysregulation (**Tables 6**, **7**).

Epigenetic co-activators CBP/p300 couple chromatin state to mitochondrial biogenesis and stress signaling by directly regulating transcriptional programs that control nuclear-encoded mitochondrial genes (283). CBP/p300 acetylates and coactivates PGC-1α and other transcription factors at enhancers/promoters of mitochondrial genes, increasing mitochondrial biogenesis and OXPHOS capacity; CBP/p300 is also required for the mitochondrial unfolded protein response, linking mitochondrial proteotoxic stress to adaptive nuclear transcriptional programs. Loss or inhibition of CBP/p300 therefore impairs mitobiogenesis, lowers OXPHOS capacity and weakens adaptive mito-nuclear signaling (123, 283–285). PRMTs modulate mitochondrial metabolism through dual routes: (1) direct substrate methylation of metabolic enzymes (for example, PRMT-mediated methylation of glycolytic or TCA cycle enzymes alters their activity or stability), and (2) chromatin/histone arginine methylation that reprograms transcriptional networks controlling mitochondrial function. By changing the balance of glycolysis versus OXPHOS, PRMT activity can shift cellular reliance on mitochondria and indirectly influence ROS generation and mitochondrial quality control. Several PRMTs have been linked to altered metabolic enzyme function and tumor metabolic phenotypes in cancer models (123, 283–285). Histone methyltransferase EZH2 (PRC2 catalytic subunit) influences mitochondrial biology largely via transcriptional repression and chromatin remodeling: EZH2-mediated H3K27me3 can silence nuclear genes encoding mitochondrial regulators (including factors that modulate lipid metabolism, mitochondrial dynamics, or antioxidant responses), thereby reprogramming cellular metabolism toward or away from oxidative phosphorylation (285). In addition, EZH2 perturbation alters ER-mitochondrial contacts and organelle homeostasis in tumor cells, changing Ca²^+^ flux, bioenergetic coupling, and ROS signaling that feedback on epigenetic enzyme activity. Thus, EZH2 inhibition may derepress mitochondrial programs (or destabilize mitochondrial ER crosstalk), altering mitochondrial respiration, redox balance and susceptibility to cell death (285). PGC-1α and mito-nuclear feedback: PGC-1α functions as a nodal integrator of many of these epigenetic inputs (acetylation by CBP/p300, deacetylation by SIRT1). Changes in CBP/p300, PRMTs or EZH2 activity converge on PGC-1α-driven transcriptional networks and on downstream factors such as NRF1/TFAM that control mtDNA transcription/replication; therefore, epigenetic modulation alters mitochondrial mass, OXPHOS capacity, and ROS output, key determinants of metastatic fitness and response to therapy (123, 284, 285).

In cancer (including CRC), these mechanistic axes predict that CBP/p300 inhibition will reduce mitobiogenesis and sensitize OXPHOS-addicted tumors, PRMT inhibition may shift metabolic flux and affect mitochondrial enzyme activity, and EZH2 inhibition can rewire mitochondrial gene expression and ER–mitochondrial homeostasis, each producing distinct changes in mtDNA copy number, oxygen consumption rate (OCR), mitochondrial membrane potential, and mtROS (123, 283–285). Key experiments to validate these links include: chromatin immunoprecipitation at nuclear-encoded mitochondrial gene loci (for CBP/p300 and EZH2), mass-spec methylome and proteome profiling (to identify PRMT substrates), functional respirometry (Seahorse OCR/ECAR), mtDNA quantification, and assays of ER–mitochondrial contact (e.g., split-GFP or electron microscopy) and future studies should explore these concepts using these significant techniques (123, 283–285).

Other targeted therapies for metastatic colorectal cancer in clinical trials: According to clinicaltrials.gov. (https://clinicaltrials.gov/), the following targeted therapies that focus on genetic mutations driving tumor growth represent a key area of research for metastatic colorectal cancer (**Tables 6**, **7**). The goal is to develop agents that inhibit the activity of abnormal proteins produced by these mutations. Encorafenib (Braftovi) targets the BRAF protein and is approved for treating patients with metastatic CRC harboring specific BRAF mutations. It is used in combination with cetuximab (Erbitux) in adults who have undergone prior treatment. Similarly, vemurafenib (Zelboraf) targets mutant B-Raf proteins and has demonstrated effectiveness in NCI-supported trials for CRC with BRAF mutations. It is administered in combination with cetuximab and irinotecan (Camptosar). The SOLARIS trial is investigating the addition of vitamin D3 to chemotherapy and bevacizumab to enhance treatment efficacy in metastatic CRC. Additionally, tucatinib (Tukysa) and trastuzumab (Herceptin) target the HER2 protein and were approved in January 2023 for advanced CRC with HER2 overexpression. In the MOUNTAINEER clinical trial, over one-third of participants experienced tumor shrinkage or disappearance, while another third achieved stable disease (**Table 7**).

Exploring combination therapies: Future research should focus on identifying optimal combinations of targeted therapies with conventional treatments to enhance efficacy and reduce resistance. Long-term efficacy and safety: Longitudinal studies are needed to evaluate the long-term efficacy and safety of these targeted therapies in diverse patient populations. Expansion to other mutations: Investigating the potential of targeted therapies against other genetic mutations in metastatic chemoresistant colorectal cancer could broaden the scope of treatment options available. By continuing to advance targeted therapy research, we can develop more effective treatments for metastatic chemoresistant colorectal cancer, ultimately improving patient outcomes.

## 8 Conclusions and future directions

The intricate relationship between mtDNA mutations and CRC metastasis describes the pivotal role of mitochondria in tumor progression. Specific mtDNA mutations can act as key drivers of CRC metastasis, potentially serving as prognostic markers and therapeutic targets for metastatic chemoresistant colorectal cancer. Variations in mitochondrial DNA copy number are closely linked to CRC progression, highlighting their potential contribution to both cancer initiation and metastasis. The exploration of mitochondrial CNVs offers insights into tumor heterogeneity and resistance mechanisms. Involvement of mitoepigenetic mechanisms, including DNA methylation and histone modifications, plays a crucial role in modulating mitochondrial gene expression in CRC. Dysregulation of these pathways contributes to both tumor growth and chemoresistance, suggesting that targeting epigenetic machinery holds promise for reversing resistance and controlling tumor progression.

### 8.1 Targeting epigenetic writer proteins in CRC

Epigenetic writer proteins, such as EZH2, EP300/CBP, and PRMTs, describe significant therapeutic targets in CRC. These enzymes alter histone methylation and acetylation, affecting gene expression programs crucial for CRC proliferation and metastasis. Selective inhibitors targeting these proteins provide new avenues for therapeutic intervention. Future research should aim to delineate the precise mechanisms by which mitochondrial DNA mutations influence cancer metastasis and chemoresistance. Detailed functional studies are required to explore how these mutations drive oncogenic pathways and alter mitochondrial function in CRC. Further investigation into the role of mitoepigenetics in tumor heterogeneity is essential. Studying how epigenetic modifications within mitochondria contribute to differential gene expression in various CRC subtypes could provide novel biomarkers for diagnosis and targeted therapy.

### 8.2 Development of epigenetic-targeted therapies

As research into epigenetic writer proteins continues to advance, the development of highly specific inhibitors targeting these proteins should be prioritized. New technologies, such as PROTACs, need to be further refined for clinical application in CRC to enhance specificity, reduce off-target effects, and improve therapeutic efficacy.

### 8.3 Integration of genomic and epigenomic data in CRC therapy

The integration of genomic and epigenomic data will be crucial in the development of personalized treatment strategies. By combining insights from mtDNA mutations, epigenetic modifications, and tumor genomics, researchers can identify key vulnerabilities in chemoresistant CRC and devise combination therapies tailored to individual tumor profiles.

### 8.4 Clinical trials and translational research

Moving forward, it is essential to translate preclinical findings into clinical trials, testing the efficacy of mitochondrial and epigenetic-targeted therapies. Collaboration between researchers, clinicians, and pharmaceutical industries will be critical to ensure the rapid development of these novel therapeutic approaches for CRC patients.

## References

[1] Zheng H-C . The molecular mechanisms of chemoresistance in cancers. Oncotarget. (2017) 8:59950. doi: 10.18632/oncotarget.19048, PMID: 28938696 PMC5601792

[2] Lichtenstein P Holm NV Verkasalo PK Iliadou A Kaprio J Koskenvuo M . Environmental and heritable factors in the causation of cancer—analyses of cohorts of twins from Sweden, Denmark, and Finland. New Engl J Med. (2000) 343:78–85. doi: 10.1056/NEJM200007133430201, PMID: 10891514

[3] Broderick P Carvajal-Carmona L Pittman AM Webb E Howarth K Rowan A . A genome-wide association study shows that common alleles of SMAD7 influence colorectal cancer risk. Nat Genet. (2007) 39:1315–7. doi: 10.1038/ng.2007.18, PMID: 17934461

[4] Zanke BW Greenwood CM Rangrej J Kustra R Tenesa A Farrington SM . Genome-wide association scan identifies a colorectal cancer susceptibility locus on chromosome 8q24. Nat Genet. (2007) 39:989–94. doi: 10.1038/ng2089, PMID: 17618283

[5] Tomlinson I Webb E Carvajal-Carmona L Broderick P Kemp Z Spain S . A genome-wide association scan of tag SNPs identifies a susceptibility variant for colorectal cancer at 8q24. 21. Nat Genet. (2007) 39:984–8. doi: 10.1038/ng2085, PMID: 17618284

[6] Tomlinson IP Webb E Carvajal-Carmona L Broderick P Howarth K Pittman AM . A genome-wide association study identifies colorectal cancer susceptibility loci on chromosomes 10p14 and 8q23. 3. Nat Genet. (2008) 40:623–30. doi: 10.1038/ng.111, PMID: 18372905

[7] Jia W-H Zhang B Matsuo K Shin A Xiang Y-B Jee SH . Genome-wide association analyses in East Asians identify new susceptibility loci for colorectal cancer. Nat Genet. (2013) 45:191–6. doi: 10.1038/ng.2505, PMID: 23263487 PMC3679924

[8] Warburg O Dickens F . The metabolism of tumors. London: Constable & Co. Ltd (1930).

[9] Warburg O . On respiratory impairment in cancer cells. Science. (1956) 124:269–70. doi: 10.1126/science.124.3215.269 13351639

[10] Pedersen PL . Tumor mitochondria and the bioenergetics of cancer cells. In: Membrane anomalies of tumor cells Basel (Switzerland): Progress in Tumor Research, Karger Publishers (1978) 22:190–274. 10.1159/000401202 149996

[11] Simonnet H Alazard N Pfeiffer K Gallou C Béroud C Demont J . Low mitochondrial respiratory chain content correlates with tumor aggressiveness in renal cell carcinoma. Carcinogenesis. (2002) 23:759–68. doi: 10.1093/carcin/23.5.759, PMID: 12016148

[12] Li Y Beckman KB Caberto C Kazma R Lum-Jones A Haiman CA . Association of genes, pathways, and haplogroups of the mitochondrial genome with the risk of colorectal cancer: the multiethnic cohort. PloS One. (2015) 10:e0136796. doi: 10.1371/journal.pone.0136796, PMID: 26340450 PMC4560485

[13] Taanman J . The mitochondrial genome: structure, transcription, translation and replication. BBA-Bioenergetics. (1999) 1410:103–23. doi: 10.1016/S0005-2728(98)00161-3, PMID: 10076021

[14] Vikram HP Kumar TP Kumar G Beeraka NM Deka R Suhail SM . Nitrosamines crisis in pharmaceuticals-insights on toxicological implications, root causes and risk assessment: a systematic review. J Pharm Anal. (2023) 14:100919. 38799236 10.1016/j.jpha.2023.12.009 PMC11126534

[15] Tyagi A Pramanik R Vishnubhatla S Ali S Bakhshi R Chopra A . Pattern of mitochondrial D-loop variations and their relation with mitochondrial encoded genes in pediatric acute myeloid leukemia. Mutat Research/Fundamental Mol Mech Mutagenesis. (2018) 810:13–8. doi: 10.1016/j.mrfmmm.2018.05.002, PMID: 29883862

[16] Sbisà E Tanzariello F Reyes A Pesole G Saccone C . Mammalian mitochondrial D-loop region structural analysis: identification of new conserved sequences and their functional and evolutionary implications. Gene. (1997) 205:125–40. doi: 10.1016/S0378-1119(97)00404-6, PMID: 9461386

[17] Coppedè F Stoccoro A . Mitoepigenetics and neurodegenerative diseases. Front Endocrinol. (2019) 10:86. doi: 10.3389/fendo.2019.00086, PMID: 30837953 PMC6389613

[18] Crimi M Sciacco M Galbiati S Bordoni A Malferrari G Bo RD . A collection of 33 novel human mtDNA homoplasmic variants. Hum Mutat. (2002) 20:409–9. doi: 10.1002/(ISSN)1098-1004, PMID: 12402350

[19] Nicholls TJ Minczuk M . In D-loop: 40 years of mitochondrial 7S DNA. Exp Gerontology. (2014) 56:175–81. doi: 10.1016/j.exger.2014.03.027, PMID: 24709344

[20] Carew JS Huang P . Mitochondrial defects in cancer. Mol Cancer. (2002) 1:1–12. doi: 10.1186/1476-4598-1-9, PMID: 12513701 PMC149412

[21] Hervouet E Simonnet H Godinot C . Mitochondria and reactive oxygen species in renal cancer. Biochimie. (2007) 89:1080–8. doi: 10.1016/j.biochi.2007.03.010, PMID: 17466430

[22] Lee HC Yin PH Lin JC Wu CC Chen CY Wu CW . Mitochondrial genome instability and mtDNA depletion in human cancers. Ann New York Acad Sci. (2005) 1042:109–22. doi: 10.1196/annals.1338.011, PMID: 15965052

[23] Thyagarajan B Wang R Barcelo H Koh W-P Yuan J-M . Mitochondrial copy number is associated with colorectal cancer risk. Cancer Epidemiology Biomarkers Prev. (2012) 21:1574–81. doi: 10.1158/1055-9965.EPI-12-0138-T, PMID: 22787200 PMC3437007

[24] Webb E Broderick P Chandler I Lubbe S Penegar S Tomlinson I . Comprehensive analysis of common mitochondrial DNA variants and colorectal cancer risk. Br J Cancer. (2008) 99:2088–93. doi: 10.1038/sj.bjc.6604805, PMID: 19050702 PMC2607223

[25] Sehgal M Ramu S Vaz JM Ganapathy YR Muralidharan S Venkatraghavan S . Characterizing heterogeneity along EMT and metabolic axes in colorectal cancer reveals underlying consensus molecular subtype-specific trends. Trans Oncol. (2024) 40:101845. doi: 10.1016/j.tranon.2023.101845, PMID: 38029508 PMC10698572

[26] Liu T Sun S Huang Y E Y Li W Xu F . The integration of single-cell and metabolomics reveals the increase of oxidative phosphorylation during the liver metastasis of colorectal cancer. Cancer Metab. (2025) 13:41. doi: 10.1186/s40170-025-00408-z, PMID: 41068982 PMC12512483

[27] Qiu X Wang A Wang J Zhang Z Tao L . Mitochondrial metabolic reprogramming in colorectal cancer: mechanisms of resistance and future clinical interventions. Cell Death Discov. (2025) 11:375. doi: 10.1038/s41420-025-02670-y, PMID: 40783392 PMC12335542

[28] Ramroop JR Gerber MM Toland AE . Germline variants impact somatic events during tumorigenesis. Trends Genet. (2019) 35:515–26. doi: 10.1016/j.tig.2019.04.005, PMID: 31128889 PMC6571050

[29] Martincorena I Raine KM Gerstung M Dawson KJ Haase K Van Loo P . Universal patterns of selection in cancer and somatic tissues. Cell. (2017) 171:1029–1041.e1021. doi: 10.1016/j.cell.2017.09.042, PMID: 29056346 PMC5720395

[30] Sun X Zhan L Chen Y Wang G He L Wang Q . Increased mtDNA copy number promotes cancer progression by enhancing mitochondrial oxidative phosphorylation in microsatellite-stable colorectal cancer. Signal Transduction Targeted Ther. (2018) 3:8. doi: 10.1038/s41392-018-0011-z, PMID: 29610678 PMC5878831

[31] Guo W Liu Y Ji X Guo S Xie F Chen Y . Mutational signature of mtDNA confers mechanistic insight into oxidative metabolism remodeling in colorectal cancer. Theranostics. (2023) 13:324. doi: 10.7150/thno.78718, PMID: 36593960 PMC9800724

[32] Khalil A Supek F . DiffInvex identifies evolutionary shifts in driver gene repertoires during tumorigenesis and chemotherapy. Nat Commun. (2025) 16:4209. doi: 10.1038/s41467-025-59397-8, PMID: 40360478 PMC12075687

[33] Qu X Hamidi H Johnson RM Sokol ES Lin E Eng C . Ligand-activated EGFR/MAPK signaling but not PI3K, are key resistance mechanisms to EGFR-therapy in colorectal cancer. Nat Commun. (2025) 16:4332. doi: 10.1038/s41467-025-59588-3, PMID: 40346041 PMC12064836

[34] Behmanesh MA Rafiee P Eidikhosh F Moridnia A . Unraveling the link between GNAS R201 mutation and colorectal cancer. Sci Rep. (2025) 15:32003. doi: 10.1038/s41598-025-17399-y, PMID: 40885811 PMC12398479

[35] Jo YS Kim MS Lee JH Lee SH An CH Yoo NJ . Frequent frameshift mutations in 2 mononucleotide repeats of RNF43 gene and its regional heterogeneity in gastric and colorectal cancers. Hum Pathol. (2015) 46:1640–6. doi: 10.1016/j.humpath.2015.07.004, PMID: 26297255

[36] Liu S-C Gong L-L Huang F-C Xu N Yang K-X Liu X-H . RNF114 facilitates the proliferation, stemness, and metastasis of colorectal cancer. Pathology-Research Pract. (2023) 248:154716. doi: 10.1016/j.prp.2023.154716, PMID: 37523804

[37] Harada A Yasumizu Y Harada T Fumoto K Sato A Maehara N . Hypoxia-induced Wnt5a-secreting fibroblasts promote colon cancer progression. Nat Commun. (2025) 16:3653. doi: 10.1038/s41467-025-58748-9, PMID: 40246836 PMC12006413

[38] Lan T-H Li W Wang X Geng Y-J Pang B Kang L-F . METTL3 promotes peritoneal metastasis of colorectal cancer through regulating m6A modification of NRXN3 mRNA. iScience. (2025) 28:113165. doi: 10.1016/j.isci.2025.113165, PMID: 40792021 PMC12337687

[39] Yapa NM Lisnyak V Reljic B Ryan MT . Mitochondrial dynamics in health and disease. FEBS Lett. (2021) 595:1184–204. doi: 10.1002/1873-3468.14077, PMID: 33742459

[40] Brown JA Sammy MJ Ballinger SW . An evolutionary, or “Mitocentric” perspective on cellular function and disease. Redox Biol. (2020) 36:101568. doi: 10.1016/j.redox.2020.101568, PMID: 32512469 PMC7281786

[41] Schon KR Ratnaike T Van Den Ameele J Horvath R Chinnery PF . Mitochondrial diseases: a diagnostic revolution. Trends Genet. (2020) 36:702–17. doi: 10.1016/j.tig.2020.06.009, PMID: 32674947

[42] Taylor RW Turnbull DM . Mitochondrial DNA mutations in human disease. Nat Rev Genet. (2005) 6:389–402. doi: 10.1038/nrg1606, PMID: 15861210 PMC1762815

[43] Zheng Y Liu J Beeraka NM Manogaran P PR HV Yn LD . Inflammation and stem cell stochasticity of HPV-induced cervical cancer: epigenetics based biomarkers through microbiome and metabolome for personalized medicine: A systematic review. Curr Medicinal Chem. (2024) 32:2390–408. doi: 10.2174/0109298673257429231108072717, PMID: 38018189

[44] Nguyen NNY Kim SS Jo YH . Deregulated mitochondrial DNA in diseases. DNA Cell Biol. (2020) 39:1385–400. doi: 10.1089/dna.2019.5220, PMID: 31944832

[45] Silva-Pinheiro P Minczuk M . The potential of mitochondrial genome engineering. Nat Rev Genet. (2022) 23:199–214. doi: 10.1038/s41576-021-00432-x, PMID: 34857922

[46] Russell O . Mitochondrial diseases: hope for the future. 四川生理科学杂志. (2021) 42:169–9.

[47] Chong M Mohammadi-Shemirani P Perrot N Nelson W Morton R Narula S . GWAS and ExWAS of blood mitochondrial DNA copy number identifies 71 loci and highlights a potential causal role in dementia. Elife. (2022) 11:e70382. doi: 10.7554/eLife.70382, PMID: 35023831 PMC8865845

[48] Fan R Zheng Y Yn LD Beeraka NM Vikram P Suhail SM . Updated intrinsic role of phytochemicals against glyphosate-induced neurotoxicity: systematic review. Curr Medicinal Chem. (2024) 32:3243–325. doi: 10.2174/0109298673257171231115114543, PMID: 38243981

[49] Durai P Beeraka NM Ramachandrappa HVP Krishnan P Gudur P Raghavendra NM . Advances in PPARs Molecular Dynamics and Glitazones as a Repurposing Therapeutic Strategy through Mitochondrial Redox Dynamics against Neurodegeneration. Curr Neuropharmacology. (2022) 20:893. doi: 10.2174/1570159X19666211109141330, PMID: 34751120 PMC9881103

[50] Gunta U Kandula DKR Gorti SKK Vadla GP Kodiyala G . Investigating the Binding Efficacy of Snake Venom Proteins as GLP-1 Analogs for Diabetes mellitus Management: An In silico Study. Oriental J Chem. (2023) 39:581–91. doi: 10.13005/ojc

[51] Singh L Atilano SR Jager MJ Kenney MC . Mitochondrial DNA polymorphisms and biogenesis genes in primary and metastatic uveal melanoma cell lines. Cancer Genet. (2021) 256:91–9. doi: 10.1016/j.cancergen.2021.05.002, PMID: 34082186

[52] Pérez-Amado CJ Bazan-Cordoba A Hidalgo-Miranda A Jiménez-Morales S . Mitochondrial heteroplasmy shifting as a potential biomarker of cancer progression. Int J Mol Sci. (2021) 22:7369. doi: 10.3390/ijms22147369, PMID: 34298989 PMC8304746

[53] Reznik E Wang Q La K Schultz N Sander C . Mitochondrial respiratory gene expression is suppressed in many cancers. Elife. (2017) 6:e21592. doi: 10.7554/eLife.21592, PMID: 28099114 PMC5243113

[54] Kalsbeek AM Chan EK Corcoran NM Hovens CM Hayes VM . Mitochondrial genome variation and prostate cancer: a review of the mutational landscape and application to clinical management. Oncotarget. (2017) 8:71342. doi: 10.18632/oncotarget.19926, PMID: 29050365 PMC5642640

[55] Reznik E Miller ML Şenbabaoğlu Y Riaz N Sarungbam J Tickoo SK . Mitochondrial DNA copy number variation across human cancers. Elife. (2016) 5:e10769. doi: 10.7554/eLife.10769, PMID: 26901439 PMC4775221

[56] Hsu C-C Tseng L-M Lee H-C . Role of mitochondrial dysfunction in cancer progression. Exp Biol Med. (2016) 241:1281–95. doi: 10.1177/1535370216641787, PMID: 27022139 PMC4950268

[57] Van Gisbergen MW Voets AM Starmans MH De Coo IF Yadak R Hoffmann RF . How do changes in the mtDNA and mitochondrial dysfunction influence cancer and cancer therapy? Challenges, opportunities and models. Mutat Research/Reviews Mutat Res. (2015) 764:16–30. doi: 10.1016/j.mrrev.2015.01.001, PMID: 26041263

[58] Chan DC . Mitochondrial dynamics and its involvement in disease. Annu Rev pathology: Mech Dis. (2020) 15:235–59. doi: 10.1146/annurev-pathmechdis-012419-032711, PMID: 31585519

[59] Zhu D Li X Tian Y . Mitochondrial-to-nuclear communication in aging: an epigenetic perspective. Trends Biochem Sci. (2022) 47:645–59. doi: 10.1016/j.tibs.2022.03.008, PMID: 35397926

[60] Matilainen O Quirós PM Auwerx J . Mitochondria and epigenetics–crosstalk in homeostasis and stress. Trends Cell Biol. (2017) 27:453–63. doi: 10.1016/j.tcb.2017.02.004, PMID: 28274652

[61] Smirnov VV Beeraka NM Butko DY Nikolenko VN Bondarev SA Achkasov EE . Updates on molecular targets and epigenetic-based therapies for PCOS. Reprod Sci. (2023) 30:772–86. doi: 10.1007/s43032-022-01013-x, PMID: 35764857

[62] Picard M Zhang J Hancock S Derbeneva O Golhar R Golik P . Progressive increase in mtDNA 3243A> G heteroplasmy causes abrupt transcriptional reprogramming. Proc Natl Acad Sci. (2014) 111:E4033–42. doi: 10.1073/pnas.1414028111, PMID: 25192935 PMC4183335

[63] Linnane A Ozawa T Marzuki S Tanaka M . Mitochondrial DNA mutations as an important contributor to ageing and degenerative diseases. Lancet. (1989) 333:642–5. doi: 10.1016/S0140-6736(89)92145-4, PMID: 2564461

[64] Szczepanowska K Trifunovic A . Origins of mtDNA mutations in ageing. Essays Biochem. (2017) 61:325–37. doi: 10.1042/EBC20160090, PMID: 28698307

[65] Sharpley MS Marciniak C Eckel-Mahan K McManus M Crimi M Waymire K . Heteroplasmy of mouse mtDNA is genetically unstable and results in altered behavior and cognition. Cell. (2012) 151:333–43. doi: 10.1016/j.cell.2012.09.004, PMID: 23063123 PMC4175720

[66] Stewart JB Alaei-Mahabadi B Sabarinathan R Samuelsson T Gorodkin J Gustafsson CM . Simultaneous DNA and RNA mapping of somatic mitochondrial mutations across diverse human cancers. PloS Genet. (2015) 11:e1005333. doi: 10.1371/journal.pgen.1005333, PMID: 26125550 PMC4488357

[67] Ju YS Alexandrov LB Gerstung M Martincorena I Nik-Zainal S Ramakrishna M . Origins and functional consequences of somatic mitochondrial DNA mutations in human cancer. Elife. (2014) 3:e02935. doi: 10.7554/eLife.02935, PMID: 25271376 PMC4371858

[68] Sercel AJ Carlson NM Patananan AN Teitell MA . Mitochondrial DNA dynamics in reprogramming to pluripotency. Trends Cell Biol. (2021) 31:311–23. doi: 10.1016/j.tcb.2020.12.009, PMID: 33422359 PMC7954944

[69] Welch DR Foster C Rigoutsos I . Roles of mitochondrial genetics in cancer metastasis. Trends Cancer. (2022) 8:1002–18. doi: 10.1016/j.trecan.2022.07.004, PMID: 35915015 PMC9884503

[70] Zhang W Bojorquez-Gomez A Velez DO Xu G Sanchez KS Shen JP . A global transcriptional network connecting noncoding mutations to changes in tumor gene expression. Nat Genet. (2018) 50:613–20. doi: 10.1038/s41588-018-0091-2, PMID: 29610481 PMC5893414

[71] Cookson W Liang L Abecasis G Moffatt M Lathrop M . Mapping complex disease traits with global gene expression. Nat Rev Genet. (2009) 10:184–94. doi: 10.1038/nrg2537, PMID: 19223927 PMC4550035

[72] Hunter KW Amin R Deasy S Ha N-H Wakefield L . Genetic insights into the morass of metastatic heterogeneity. Nat Rev Cancer. (2018) 18:211–23. doi: 10.1038/nrc.2017.126, PMID: 29422598 PMC6290469

[73] Pérez-Amado CJ Tovar H Gómez-Romero L Beltrán-Anaya FO Bautista-Piña V Dominguez-Reyes C . Mitochondrial DNA mutation analysis in breast cancer: shifting from germline heteroplasmy toward homoplasmy in tumors. Front Oncol. (2020) 10:572954. doi: 10.3389/fonc.2020.572954, PMID: 33194675 PMC7653098

[74] Scheid AD Beadnell TC Welch DR . The second genome: Effects of the mitochondrial genome on cancer progression. In: Advances in cancer research, vol. 142. USA: Elsevier (2019). p. 63–105., PMID: 30885364 10.1016/bs.acr.2019.01.001 PMC6921473

[75] Scheid AD Beadnell TC Welch DR . Roles of mitochondria in the hallmarks of metastasis. Br J Cancer. (2021) 124:124–35. doi: 10.1038/s41416-020-01125-8, PMID: 33144695 PMC7782743

[76] Yuan Y Ju YS Kim Y Li J Wang Y Yoon CJ . Comprehensive molecular characterization of mitochondrial genomes in human cancers. Nat Genet. (2020) 52:342–52. doi: 10.1038/s41588-019-0557-x, PMID: 32024997 PMC7058535

[77] Wang P Castellani CA Yao J Huan T Bielak LF Zhao W . Epigenome-wide association study of mitochondrial genome copy number. Hum Mol Genet. (2022) 31:309–19. doi: 10.1093/hmg/ddab240, PMID: 34415308 PMC8742999

[78] Zhao J Ren R Beeraka NM Mahesh P Xue N Lu P . Correlation of time trends of air pollutants, greenspaces and tracheal, bronchus and lung cancer incidence and mortality among the adults in United States. Front Oncol. (2024) 14. doi: 10.3389/fonc.2024.1398679, PMID: 39119087 PMC11306054

[79] Liu W Beck BH Vaidya KS Nash KT Feeley KP Ballinger SW . Metastasis suppressor KISS1 seems to reverse the Warburg effect by enhancing mitochondrial biogenesis. Cancer Res. (2014) 74:954–63. doi: 10.1158/0008-5472.CAN-13-1183, PMID: 24351292 PMC3946400

[80] Aaltonen L Johns L Järvinen H Mecklin J-P Houlston R . Explaining the familial colorectal cancer risk associated with mismatch repair (MMR)-deficient and MMR-stable tumors. Clin Cancer Res. (2007) 13:356–61. doi: 10.1158/1078-0432.CCR-06-1256, PMID: 17200375

[81] Tenesa A Farrington SM Prendergast JG Porteous ME Walker M Haq N . Genome-wide association scan identifies a colorectal cancer susceptibility locus on 11q23 and replicates risk loci at 8q24 and 18q21. Nat Genet. (2008) 40:631–7. doi: 10.1038/ng.133, PMID: 18372901 PMC2778004

[82] Wallace DC . A mitochondrial paradigm of metabolic and degenerative diseases, aging, and cancer: a dawn for evolutionary medicine. Annu Rev Genet. (2005) 39:359–407. doi: 10.1146/annurev.genet.39.110304.095751, PMID: 16285865 PMC2821041

[83] Benhar M Engelberg D Levitzki A . ROS, stresskigr7.et kinases and stress signaling in cancer. EMBO Rep. (2002) 3:420–5. doi: 10.1093/embo-reports/kvf094, PMID: 11991946 PMC1084107

[84] Beeraka NM Zhang J Uthaiah CA Anastasia CA Liu J Ramachandrappa HVP . Expression patterns and relevance of FN3K, nrf2, and NQO1 in breast cancers. (2024) 8:88–105. doi: 10.14744/ejmo.2024.61033

[85] Lowell BB Shulman GI . Mitochondrial dysfunction and type 2 diabetes. Science. (2005) 307:384–7. doi: 10.1126/science.1104343, PMID: 15662004

[86] Schapira A . Mitochondrial involvement in Parkinson’s disease, Huntington’s disease, hereditary spastic paraplegia and Friedreich’s ataxia. Biochim Biophys Acta (BBA)-Bioenergetics. (1999) 1410:159–70. doi: 10.1016/S0005-2728(98)00164-9, PMID: 10076024

[87] Burdon RH . Superoxide and hydrogen peroxide in relation to mammalian cell proliferation. Free Radical Biol Med. (1995) 18:775–94. doi: 10.1016/0891-5849(94)00198-S, PMID: 7750801

[88] Sruthi A Faizan S Vikram H Veena N Susil A Harindranath H . A multifaceted approach for the development of novel Hantzsch 1, 4-dihydropyridines as anticancer agents: Rational design, parallel synthesis, analysis, cytotoxicity and EGFR/HER2 inhibition studies. Results Chem. (2024) 7:101413. doi: 10.1016/j.rechem.2024.101413

[89] Beeraka NM Zhang J Zhao D Liu J Au C Vikram P . Combinatorial implications of nrf2 inhibitors with FN3K inhibitor: *in vitro* breast cancer study. Curr Pharm Design. (2023) 29:2408–25. doi: 10.2174/0113816128261466231011114600, PMID: 37861038

[90] Chatterjee A Mambo E Sidransky D . Mitochondrial DNA mutations in human cancer. Oncogene. (2006) 25:4663–74. doi: 10.1038/sj.onc.1209604, PMID: 16892080

[91] Bai R-K Leal SM Covarrubias D Liu A Wong L-JC . Mitochondrial genetic background modifies breast cancer risk. Cancer Res. (2007) 67:4687–94. doi: 10.1158/0008-5472.CAN-06-3554, PMID: 17510395

[92] Cano D Gomez C Ospina N Cajigas J Groot H Andrade R . Mitochondrial DNA haplogroups and susceptibility to prostate cancer in a Colombian population. Int Scholarly Res Notices. (2014) 2014:530675. doi: 10.1155/2014/530675, PMID: 24616820 PMC3927756

[93] Hu S-P Du J-P Li D-R Yao Y-G . Mitochondrial DNA haplogroup confers genetic susceptibility to nasopharyngeal carcinoma in Chaoshanese from Guangdong, China. PloS One. (2014) 9:e87795. doi: 10.1371/journal.pone.0087795, PMID: 24498198 PMC3909237

[94] Fang H Shen L Chen T He J Ding Z Wei J . Cancer type-specific modulation of mitochondrial haplogroups in breast, colorectal and thyroid cancer. BMC Cancer. (2010) 10:1–10. doi: 10.1186/1471-2407-10-421, PMID: 20704735 PMC2933623

[95] Ruiz-Pesini E Mishmar D Brandon M Procaccio V Wallace DC . Effects of purifying and adaptive selection on regional variation in human mtDNA. Science. (2004) 303:223–6. doi: 10.1126/science.1088434, PMID: 14716012

[96] Lim SW Kim HR Kim HY Huh JW Kim YJ Shin JH . Highgim223-226 minisatellite instability of the mitochondrial genome in colorectal cancer tissue associated with clinicopathological values. Int J Cancer. (2012) 131:1332–41. doi: 10.1002/ijc.v131.6, PMID: 22120612

[97] Boroughs LK DeBerardinis RJ . Metabolic pathways promoting cancer cell survival and growth. Nat Cell Biol. (2015) 17:351–9. doi: 10.1038/ncb3124, PMID: 25774832 PMC4939711

[98] Dang CV . Rethinking the Warburg effect with Myc micromanaging glutamine metabolism. Cancer Res. (2010) 70:859–62. doi: 10.1158/0008-5472.CAN-09-3556, PMID: 20086171 PMC2818441

[99] Cantley LC Auger KR Carpenter C Duckworth B Graziani A Kapeller R . Oncogenes and signal transduction. Cell. (1991) 64:281–302. doi: 10.1016/0092-8674(91)90639-G, PMID: 1846320

[100] Basu S Gnanapradeepan K Barnoud T Kung C-P Tavecchio M Scott J . Mutant p53 controls tumor metabolism and metastasis by regulating PGC-1α. Genes Dev. (2018) 32:230–43. doi: 10.1101/gad.309062.117, PMID: 29463573 PMC5859965

[101] Wang D Tian J Yan Z Yuan Q Wu D Liu X . Mitochondrial fragmentation is crucial for c-Myc-driven hepatoblastoma-like liver tumors. Mol Ther. (2022) 30:1645–60. doi: 10.1016/j.ymthe.2022.01.032, PMID: 35085814 PMC9077476

[102] Mihaylova VT Bindra RS Yuan J Campisi D Narayanan L Jensen R . Decreased expression of the DNA mismatch repair gene Mlh1 under hypoxic stress in mammalian cells. Mol Cell Biol. (2003) 23:3265–73. doi: 10.1128/MCB.23.9.3265-3273.2003, PMID: 12697826 PMC153206

[103] Bindra RS Crosby ME Glazer PM . Regulation of DNA repair in hypoxic cancer cells. Cancer Metastasis Rev. (2007) 26:249–60. doi: 10.1007/s10555-007-9061-3, PMID: 17415527

[104] Nakamura H Tanimoto K Hiyama K Yunokawa M Kawamoto T Kato Y . Human mismatch repair gene, MLH1, is transcriptionally repressed by the hypoxia-inducible transcription factors, DEC1 and DEC2. Oncogene. (2008) 27:4200–9. doi: 10.1038/onc.2008.58, PMID: 18345027

[105] Kujoth GC Prolla TA . Evolving insight into the role of mitochondrial DNA mutations in aging. Exp Gerontology. (2008) 43:20–3. doi: 10.1016/j.exger.2007.09.010, PMID: 18054193

[106] Feng S Xiong L Ji Z Cheng W Yang H . Correlation between increased ND2 expression and demethylated displacement loop of mtDNA in colorectal cancer. Mol Med Rep. (2012) 6:125–30., PMID: 22505229 10.3892/mmr.2012.870

[107] Mitchell SL Goodloe R Brown-Gentry K Pendergrass SA Murdock DG Crawford DC . Characterization of mitochondrial haplogroups in a large population-based sample from the United States. Hum Genet. (2014) 133:861–8. doi: 10.1007/s00439-014-1421-9, PMID: 24488180 PMC4113317

[108] Ruiz-Pesini E Lott MT Procaccio V Poole JC Brandon MC Mishmar D . An enhanced MITOMAP with a global mtDNA mutational phylogeny. Nucleic Acids Res. (2007) 35:D823–8. doi: 10.1093/nar/gkl927, PMID: 17178747 PMC1781213

[109] Van Oven M Kayser M . Updated comprehensive phylogenetic tree of global human mitochondrial DNA variation. Hum Mutat. (2009) 30:E386–94. doi: 10.1002/humu.20921, PMID: 18853457

[110] Herrnstadt C Elson JL Fahy E Preston G Turnbull DM Anderson C . Reduced-median-network analysis of complete mitochondrial DNA coding-region sequences for the major African, Asian, and European haplogroups. Am J Hum Genet. (2002) 70:1152–71. doi: 10.1086/339933, PMID: 11938495 PMC447592

[111] Wu MC Lee S Cai T Li Y Boehnke M Lin X . Rare-variant association testing for sequencing data with the sequence kernel association test. Am J Hum Genet. (2011) 89:82–93. doi: 10.1016/j.ajhg.2011.05.029, PMID: 21737059 PMC3135811

[112] Lee S Wu MC Lin X . Optimal tests for rare variant effects in sequencing association studies. Biostatistics. (2012) 13:762–75. doi: 10.1093/biostatistics/kxs014, PMID: 22699862 PMC3440237

[113] Ionita-Laza I Lee S Makarov V Buxbaum JD Lin X . Sequence kernel association tests for the combined effect of rare and common variants. Am J Hum Genet. (2013) 92:841–53. doi: 10.1016/j.ajhg.2013.04.015, PMID: 23684009 PMC3675243

[114] Wu MC Kraft P Epstein MP Taylor DM Chanock SJ Hunter DJ . Powerful SNP-set analysis for case-control genome-wide association studies. Am J Hum Genet. (2010) 86:929–42. doi: 10.1016/j.ajhg.2010.05.002, PMID: 20560208 PMC3032061

[115] Ishikawa K Takenaga K Akimoto M Koshikawa N Yamaguchi A Imanishi H . ROS-generating mitochondrial DNA mutations can regulate tumor cell metastasis. Science. (2008) 320:661–4. doi: 10.1126/science.1156906, PMID: 18388260

[116] Ishikawa K Hayashi JI . A novel function of mtDNA: its involvement in metastasis. Ann New York Acad Sci. (2010) 1201:40–3. doi: 10.1111/j.1749-6632.2010.05616.x, PMID: 20649537

[117] Imanishi H Hattori K Wada R Ishikawa K Fukuda S Takenaga K . Mitochondrial DNA mutations regulate metastasis of human breast cancer cells. PloS One. (2011) 6:e23401. doi: 10.1371/journal.pone.0023401, PMID: 21853128 PMC3154938

[118] Koshikawa N Akimoto M Hayashi J-I Nagase H Takenaga K . Association of predicted pathogenic mutations in mitochondrial ND genes with distant metastasis in NSCLC and colon cancer. Sci Rep. (2017) 7:15535. doi: 10.1038/s41598-017-15592-2, PMID: 29138417 PMC5686070

[119] Yuan Y Wang W Li H Yu Y Tao J Huang S . Nonsense and missense mutation of mitochondrial ND6 gene promotes cell migration and invasion in human lung adenocarcinoma. BMC Cancer. (2015) 15:1–10. doi: 10.1186/s12885-015-1349-z, PMID: 25934296 PMC4425906

[120] Tang S Batra A Zhang Y Ebenroth ES Huang T . Left ventricular noncompaction is associated with mutations in the mitochondrial genome. Mitochondrion. (2010) 10:350–7. doi: 10.1016/j.mito.2010.02.003, PMID: 20211276

[121] Ji Y-C Liu X-L Zhao F-X Zhang J-J Zhang Y Zhou X-T . The mitochondrial ND5 T12338C mutation may be associated with Leber's hereditary optic neuropathy in two Chinese families. Yi Chuan= Hereditas. (2011) 33:322–8. doi: 10.3724/SP.J.1005.2011.00322, PMID: 21482521

[122] Kenny T Hart P Ragazzi M Sersinghe M Chipuk J Sagar MAK . Selected mitochondrial DNA landscapes activate the SIRT3 axis of the UPRmt to promote metastasis. Oncogene. (2017) 36:4393–404. doi: 10.1038/onc.2017.52, PMID: 28368421 PMC5542861

[123] LeBleu VS O’Connell JT Gonzalez Herrera KN Wikman H Pantel K Haigis MC . PGC-1α mediates mitochondrial biogenesis and oxidative phosphorylation in cancer cells to promote metastasis. Nat Cell Biol. (2014) 16:992–1003. doi: 10.1038/ncb3039, PMID: 25241037 PMC4369153

[124] Goh J Enns L Fatemie S Hopkins H Morton J Pettan-Brewer C . Mitochondrial targeted catalase suppresses invasive breast cancer in mice. BMC Cancer. (2011) 11:1–12. doi: 10.1186/1471-2407-11-191, PMID: 21605372 PMC3123323

[125] Fatemie S Goh J Pettan-Brewer C Ladiges W . Breast tumors in PyMT transgenic mice expressing mitochondrial catalase have decreased labeling for macrophages and endothelial cells. Pathobiology Aging Age-Related Dis. (2012) 2:17391. doi: 10.3402/pba.v2i0.17391, PMID: 22953034 PMC3417526

[126] Akouchekian M Houshmand M Hemati S Ansaripour M Shafa M . High rate of mutation in mitochondrial DNA displacement loop region in human colorectal cancer. Dis Colon Rectum. (2009) 52:526–30. doi: 10.1007/DCR.0b013e31819acb99, PMID: 19333057

[127] Basso D Navaglia F Fogar P Zambon C-F Greco E Schiavon S . DNA repair pathways and mitochondrial DNA mutations in gastrointestinal carcinogenesis. Clinica Chimica Acta. (2007) 381:50–5. doi: 10.1016/j.cca.2007.02.020, PMID: 17397816

[128] Wei Y-H Lee H-C . Oxidative stress, mitochondrial DNA mutation, and impairment of antioxidant enzymes in aging. Exp Biol Med. (2002) 227:671–82. doi: 10.1177/153537020222700901, PMID: 12324649

[129] Jakupciak JP Wang W Markowitz ME Ally D Coble M Srivastava S . Mitochondrial DNA as a cancer biomarker. J Mol Diagnostics. (2005) 7:258–67. doi: 10.1016/S1525-1578(10)60553-3, PMID: 15858150 PMC1867534

[130] Kagan J Srivastava S . Mitochondria as a target for early detection and diagnosis of cancer. Crit Rev Clin Lab Sci. (2005) 42:453–72. doi: 10.1080/10408360500295477, PMID: 16390681

[131] Parr RL Dakubo GD Thayer RE McKenney K Birch-Machin MA . Mitochondrial DNA as a potential tool for early cancer detection. Hum Genomics. (2006) 2:1–6. 10.1186/1479-7364-2-4-252 PMC3500203 16460650

[132] Cavalli LR Liang BC . Mutagenesis, tumorigenicity, and apoptosis: are the mitochondria involved? Mutat Research/Fundamental Mol Mech Mutagenesis. (1998) 398:19–26., PMID: 9626961 10.1016/s0027-5107(97)00223-6

[133] Jackson AL Loeb LA . The contribution of endogenous sources of DNA damage to the multiple mutations in cancer. Mutat Research/Fundamental Mol Mech Mutagenesis. (2001) 477:7–21. doi: 10.1016/S0027-5107(01)00091-4, PMID: 11376682

[134] Kang D-H . Oxidative stress, DNA damage, and breast cancer. AACN Advanced Crit Care. (2002) 13:540–9. doi: 10.1097/00044067-200211000-00007, PMID: 12473916

[135] Alonso A Martin P Albarran C Aguilera B Garcia O Guzman A . Detection of somatic mutations in the mitochondrial DNA control region of colorectal and gastric tumors by heteroduplex and singledstrand conformation analysis. Electrophoresis. (1997) 18:682–5. doi: 10.1002/elps.1150180504, PMID: 9194590

[136] Suzuki M Toyooka S Miyajima K Iizasa T Fujisawa T Bekele NB . Alterations in the mitochondrial displacement loop in lung cancers. Clin Cancer Res. (2003) 9:5636–41., PMID: 14654546

[137] Okochi O Hibi K Uemura T Inoue S Takeda S Kaneko T . Detection of mitochondrial DNA alterations in the serum of hepatocellular carcinoma patients. Clin Cancer Res. (2002) 8:2875–8., PMID: 12231530

[138] Rosson D Keshgegian AA . Frequent mutations in the mitochondrial control region DNA in breast tissue. Cancer Lett. (2004) 215:89–94. doi: 10.1016/j.canlet.2004.04.030, PMID: 15374637

[139] Sui G Zhou S Wang J Canto M Lee EE Eshleman JR . Mitochondrial DNA mutations in preneoplastic lesions of the gastrointestinal tract: a biomarker for the early detection of cancer. Mol Cancer. (2006) 5:1–9. doi: 10.1186/1476-4598-5-73, PMID: 17166268 PMC1764424

[140] Parsons TJ Muniec DS Sullivan K Woodyatt N Alliston-Greiner R Wilson MR . A high observed substitution rate in the human mitochondrial DNA control region. Nat Genet. (1997) 15:363–8. doi: 10.1038/ng0497-363, PMID: 9090380

[141] Tamura G Nishizuka S Maesawa C Suzuki Y Iwaya T Sakata K . Mutations in mitochondrial control region DNA in gastric tumours of Japanese patients. Eur J Cancer. (1999) 35:316–9. doi: 10.1016/S0959-8049(98)00360-8, PMID: 10448277

[142] Habano W Sugai T Nakamura SI Uesugi N Yoshida T Sasou S . Microsatellite instability and mutation of mitochondrial and nuclear DNA in gastric carcinoma. Gastroenterology. (2000) 118:835–41. doi: 10.1016/S0016-5085(00)70169-7, PMID: 10784582

[143] Zickermann V Wirth C Nasiri H Siegmund K Schwalbe H Hunte C . Mechanistic insight from the crystal structure of mitochondrial complex I. Science. (2015) 347:44–9. doi: 10.1126/science.1259859, PMID: 25554780

[144] Vinothkumar KR Zhu J Hirst J . Architecture of mammalian respiratory complex I. Nature. (2014) 515:80–4. doi: 10.1038/nature13686, PMID: 25209663 PMC4224586

[145] Lightowlers RN Taylor RW Turnbull DM . Mutations causing mitochondrial disease: What is new and what challenges remain? Science. (2015) 349:1494–9. doi: 10.1126/science.aac7516, PMID: 26404827

[146] Zong W-X Rabinowitz JD White E . Mitochondria and cancer. Mol Cell. (2016) 61:667–76. doi: 10.1016/j.molcel.2016.02.011, PMID: 26942671 PMC4779192

[147] Hashizume O Shimizu A Yokota M Sugiyama A Nakada K Miyoshi H . Specific mitochondrial DNA mutation in mice regulates diabetes and lymphoma development. Proc Natl Acad Sci. (2012) 109:10528–33. doi: 10.1073/pnas.1202367109, PMID: 22689997 PMC3387115

[148] Dasgupta S Soudry E Mukhopadhyay N Shao C Yee J Lam S . Mitochondrial DNA mutations in respiratory complexto in neverextorya lung cancer patients contribute to lung cancer progression and associated with EGFR gene mutation. J Cell Physiol. (2012) 227:2451–60. doi: 10.1002/jcp.v227.6, PMID: 21830212 PMC3256258

[149] Kulawiec M Owens KM Singh KK . mtDNA G10398A variant in African-American women with breast cancer provides resistance to apoptosis and promotes metastasis in mice. J Hum Genet. (2009) 54:647–54. doi: 10.1038/jhg.2009.89, PMID: 19763141 PMC2909846

[150] Santidrian AF Matsuno-Yagi A Ritland M Seo BB LeBoeuf SE Gay LJ . Mitochondrial complex I activity and NAD+/NADH balance regulate breast cancer progression. J Clin Invest. (2013) 123:1068–81. doi: 10.1172/JCI64264, PMID: 23426180 PMC3582128

[151] Kim J-W Tchernyshyov I Semenza GL . Dang CV: HIF-1-mediated expression of pyruvate dehydrogenase kinase: a metabolic switch required for cellular adaptation to hypoxia. Cell Metab. (2006) 3:177–85. doi: 10.1016/j.cmet.2006.02.002, PMID: 16517405

[152] Masoud GN Li W . HIF-1α pathway: role, regulation and intervention for cancer therapy. Acta Pharm Sin B. (2015) 5:378–89. doi: 10.1016/j.apsb.2015.05.007, PMID: 26579469 PMC4629436

[153] Su S-C Lin C-W Yang W-E Fan W-L Yang S-F . The urokinase-type plasminogen activator (uPA) system as a biomarker and therapeutic target in human Malignancies. Expert Opin Ther Targets. (2016) 20:551–66. doi: 10.1517/14728222.2016.1113260, PMID: 26667094

[154] Zhang X Huang S Guo J Zhou L You L Zhang T . Insights into the distinct roles of MMP-11 in tumor biology and future therapeutics. Int J Oncol. (2016) 48:1783–93. doi: 10.3892/ijo.2016.3400, PMID: 26892540

[155] Su B Zhao W Shi B Zhang Z Yu X Xie F . Let-7d suppresses growth, metastasis, and tumor macrophage infiltration in renal cell carcinoma by targeting COL3A1 and CCL7. Mol Cancer. (2014) 13:1–13. doi: 10.1186/1476-4598-13-206, PMID: 25193015 PMC4168121

[156] Lee YS Kim S-Y Song SJ Hong HK Lee Y Oh BY . Crosstalk between CCL7 and CCR3 promotes metastasis of colon cancer cells via ERK-JNK signaling pathways. Oncotarget. (2016) 7:36842. doi: 10.18632/oncotarget.v7i24, PMID: 27167205 PMC5095043

[157] Hao N-B Lü M-H Fan Y-H Cao Y-L Zhang Z-R Yang S-M . Macrophages in tumor microenvironments and the progression of tumors. J Immunol Res. (2012) 2012:948098. doi: 10.1155/2012/948098, PMID: 22778768 PMC3385963

[158] Prenen H Tejpar S Cutsem EV . New strategies for treatment of KRAS mutant metastatic colorectal cancer. Clin Cancer Res. (2010) 16:2921–6. doi: 10.1158/1078-0432.CCR-09-2029, PMID: 20460490

[159] Wolfer A Ramaswamy S . MYC and metastasis. Cancer Res. (2011) 71:2034–7. doi: 10.1158/0008-5472.CAN-10-3776, PMID: 21406394 PMC3089000

[160] Rapp UR Korn C Ceteci F Karreman C Luetkenhaus K Serafin V . MYC is a metastasis gene for non-small-cell lung cancer. PloS One. (2009) 4:e6029. doi: 10.1371/journal.pone.0006029, PMID: 19551151 PMC2696940

[161] Orian-Rousseau V . CD44 acts as a signaling platform controlling tumor progression and metastasis. Front Immunol. (2015) 6:154. doi: 10.3389/fimmu.2015.00154, PMID: 25904917 PMC4389564

[162] Ishikawa K Hashizume O Koshikawa N Fukuda S Nakada K Takenaga K . Enhanced glycolysis induced by mtDNA mutations does not regulate metastasis. FEBS Lett. (2008) 582:3525–30. doi: 10.1016/j.febslet.2008.09.024, PMID: 18805414

[163] Vinogradov AD Grivennikova VG . Oxidation of NADH and ROS production by respiratory complex I. Biochim Biophys Acta (BBA)-Bioenergetics. (2016) 1857:863–71. doi: 10.1016/j.bbabio.2015.11.004, PMID: 26571336

[164] Bleier L Dröse S . Superoxide generation by complex III: from mechanistic rationales to functional consequences. Biochim Biophys Acta (BBA)-Bioenergetics. (2013) 1827:1320–31. doi: 10.1016/j.bbabio.2012.12.002, PMID: 23269318

[165] Moloney JN Cotter TG . ROS signalling in the biology of cancer. In: Seminars in cell & developmental biology, vol. 2018. USA: Elsevier (2018). p. 50–64. 10.1016/j.semcdb.2017.05.023 28587975

[166] Fassone E Rahman S . Complex I deficiency: clinical features, biochemistry and molecular genetics. J Med Genet. (2012) 49:578–90. doi: 10.1136/jmedgenet-2012-101159, PMID: 22972949

[167] Rodenburg RJ . Mitochondrial complex I-linked disease. Biochim Biophys Acta (BBA)-Bioenergetics. (2016) 1857:938–45. doi: 10.1016/j.bbabio.2016.02.012, PMID: 26906428

[168] Lu J Tan M Cai Q . The Warburg effect in tumor progression: mitochondrial oxidative metabolism as an anti-metastasis mechanism. Cancer Lett. (2015) 356:156–64. doi: 10.1016/j.canlet.2014.04.001, PMID: 24732809 PMC4195816

[169] Zheng J . Energy metabolism of cancer: Glycolysis versus oxidative phosphorylation. Oncol Lett. (2012) 4:1151–7. doi: 10.3892/ol.2012.928, PMID: 23226794 PMC3506713

[170] Wu CW Yin PH Hung WY Li AFY Li SH Chi CW . Mitochondrial DNA mutations and mitochondrial DNA depletion in gastric cancer. Genes Chromosomes Cancer. (2005) 44:19–28. doi: 10.1002/gcc.20213, PMID: 15892105

[171] Mambo E Chatterjee A Xing M Tallini G Haugen BR Yeung SCJ . Tumornskyeomos changes in mtDNA content in human cancer. Int J Cancer. (2005) 116:920–4. doi: 10.1002/ijc.v116:6 15856456

[172] Yin P Lee H Chau G Wu Y Li S Lui W . Alteration of the copy number and deletion of mitochondrial DNA in human hepatocellular carcinoma. Br J Cancer. (2004) 90:2390–6. doi: 10.1038/sj.bjc.6601838, PMID: 15150555 PMC2409531

[173] Lin C-S Wang L-S Tsai C-M Wei Y-H . Low copy number and low oxidative damage of mitochondrial DNA are associated with tumor progression in lung cancer tissues after neoadjuvant chemotherapy. Interactive Cardiovasc Thorac Surg. (2008) 7:954–8. doi: 10.1510/icvts.2008.177006, PMID: 18685121

[174] Meierhofer D Mayr JA Foetschl U Berger A Fink K Schmeller N . Decrease of mitochondrial DNA content and energy metabolism in renal cell carcinoma. Carcinogenesis. (2004) 25:1005–10. doi: 10.1093/carcin/bgh104, PMID: 14764459

[175] Guo J Zheng L Liu W Wang X Wang Z Wang Z . Frequent truncating mutation of TFAM induces mitochondrial DNA depletion and apoptotic resistance in microsatellite-unstable colorectal cancer. Cancer Res. (2011) 71:2978–87. doi: 10.1158/0008-5472.CAN-10-3482, PMID: 21467167 PMC3710668

[176] Egan K Kusao I Troelstrup D Agsalda M Shiramizu B . Mitochondrial DNA in residual leukemia cells in cerebrospinal fluid in children with acute lymphoblastic leukemia. J Clin Med Res. (2010) 2:225. doi: 10.4021/jocmr443w, PMID: 21331151 PMC3039488

[177] Lin C-S Chang S-C Wang L-S Chou T-Y Hsu W-H Wu Y-C . The role of mitochondrial DNA alterations in esophageal squamous cell carcinomas. J Thorac Cardiovasc Surg. (2010) 139:189–197.e184. doi: 10.1016/j.jtcvs.2009.04.007, PMID: 19660406

[178] Kim MM Clinger JD Masayesva BG Ha PK Zahurak ML Westra WH . Mitochondrial DNA quantity increases with histopathologic grade in premalignant and Malignant head and neck lesions. Clin Cancer Res. (2004) 10:8512–5. doi: 10.1158/1078-0432.CCR-04-0734, PMID: 15623632

[179] Wang Y Liu V Xue W Cheung A Ngan H . Association of decreased mitochondrial DNA content with ovarian cancer progression. Br J Cancer. (2006) 95:1087–91. doi: 10.1038/sj.bjc.6603377, PMID: 17047655 PMC2360719

[180] Mizumachi T Muskhelishvili L Naito A Furusawa J Fan CY Siegel ER . Increased distributional variance of mitochondrial DNA content associated with prostate cancer cells as compared with normal prostate cells. Prostate. (2008) 68:408–17. doi: 10.1002/pros.20697, PMID: 18196528 PMC2268637

[181] Yu M . Generation, function and diagnostic value of mitochondrial DNA copy number alterations in human cancers. Life Sci. (2011) 89:65–71. doi: 10.1016/j.lfs.2011.05.010, PMID: 21683715

[182] Feng S Xiong L Ji Z Cheng W Yang H . Correlation between increased copy number of mitochondrial DNA and clinicopathological stage in colorectal cancer. Oncol Lett. (2011) 2:899–903. 22866147 10.3892/ol.2011.322 PMC3408021

[183] Wen S Gao J Zhang L Zhou H Fang D Feng S . p53 increase mitochondrial copy number via up-regulation of mitochondrial transcription factor A in colorectal cancer. Oncotarget. (2016) 7:75981. doi: 10.18632/oncotarget.12514, PMID: 27732955 PMC5342792

[184] Hayashi J-I Takemitsu M Nonaka I . Recovery of the missing tumorigenicity in mitochondrial DNA-less HeLa cells by introduction of mitochondrial DNA from normal human cells. Somatic Cell Mol Genet. (1992) 18:123–9. doi: 10.1007/BF01233159, PMID: 1574738

[185] Mizumachi T Suzuki S Naito A Carcel-Trullols J Evans T Spring P . Increased mitochondrial DNA induces acquired docetaxel resistance in head and neck cancer cells. Oncogene. (2008) 27:831–8. doi: 10.1038/sj.onc.1210681, PMID: 17637738 PMC2268644

[186] Xu Y Lu S . Transforming growth factor−β1−induced epithelial to mesenchymal transition increases mitochondrial content in the A549 non−small cell lung cancer cell line. Mol Med Rep. (2015) 11:417–21. doi: 10.3892/mmr.2014.2678, PMID: 25323156

[187] Huang Q Li J Xing J Li W Li H Ke X . CD147 promotes reprogramming of glucose metabolism and cell proliferation in HCC cells by inhibiting the p53-dependent signaling pathway. J Hepatol. (2014) 61:859–66. doi: 10.1016/j.jhep.2014.04.035, PMID: 24801417

[188] Boland CR Goel A . Microsatellite instability in colorectal cancer. Gastroenterology. (2010) 138:2073–2087.e2073. doi: 10.1053/j.gastro.2009.12.064, PMID: 20420947 PMC3037515

[189] Choudhury AR Singh KK . Mitochondrial determinants of cancer health disparities. In: Seminars in cancer biology, vol. 2017. USA: Elsevier (2017). p. 125–46., PMID: 28487205 10.1016/j.semcancer.2017.05.001 PMC5673596

[190] Wei Z Su W Lou H Duan S Chen G . Trafficking pathway between plasma membrane and mitochondria via clathrin-mediated endocytosis. J Mol Cell Biol. (2018) 10:539–48. doi: 10.1093/jmcb/mjy060, PMID: 30383243

[191] Cui Y Wang Y Liu M Qiu L Xing P Wang X . Determination of glucose deficiency-induced cell death by mitochondrial ATP generation-driven proton homeostasis. J Mol Cell Biol. (2017) 9:395–408. doi: 10.1093/jmcb/mjx011, PMID: 28369514

[192] Kwak S-Y Yoo J-O An H-J Bae I-H Park M-J Kim J . miR-5003-3p promotes epithelial-mesenchymal transition in breast cancer cells through Snail stabilization and direct targeting of E-cadherin. J Mol Cell Biol. (2016) 8:372–83. doi: 10.1093/jmcb/mjw026, PMID: 27282406

[193] Duarte FV Palmeira CM Rolo AP . The role of microRNAs in mitochondria: small players acting wide. Genes. (2014) 5:865–86. doi: 10.3390/genes5040865, PMID: 25264560 PMC4276918

[194] Anand P Koleto M Kandula DR Xiong L MacNeill R . Novel hydrophilic-phase extraction, HILIC and high-resolution MS quantification of an RNA oligonucleotide in plasma. Bioanalysis. (2022) 14:47–62. doi: 10.4155/bio-2021-0216, PMID: 34779651

[195] Wallace L Cherian AM Adamson P Bari S Banerjee S Flood M . Comparison of pre-and post-translational expressions of COXIV-1 and MT-ATPase 6 genes in colorectal adenoma-carcinoma tissues. J Carcinogenesis Mutagenesis. (2018) 9:319. doi: 10.4172/2157-2518.1000319, PMID: 30393577 PMC6214464

[196] Zhang X Schulze PC . MicroRNAs in heart failure: Non-coding regulators of metabolic function. Biochim Biophys Acta (BBA)-Molecular Basis Dis. (2016) 1862:2276–87. doi: 10.1016/j.bbadis.2016.08.009, PMID: 27544699 PMC5376502

[197] Chen Z Li Y Zhang H Huang P Luthra R . Hypoxia-regulated microRNA-210 modulates mitochondrial function and decreases ISCU and COX10 expression. Oncogene. (2010) 29:4362–8. doi: 10.1038/onc.2010.193, PMID: 20498629

[198] Qu A Du L Yang Y Liu H Li J Wang L . Hypoxia-inducible MiR-210 is an independent prognostic factor and contributes to metastasis in colorectal cancer. PloS One. (2014) 9:e90952. doi: 10.1371/journal.pone.0090952, PMID: 24632577 PMC3954583

[199] Kim NH Cha YH Eun Kang S Mi Lee Y Lee I Young Cha S . p53 regulates nuclear GSK-3 levels through miR-34-mediated Axin2 suppression in colorectal cancer cells. Cell Cycle. (2013) 12:1578–87. doi: 10.4161/cc.24739, PMID: 23624843 PMC3680537

[200] Nagel R le Sage C Diosdado B Van Der Waal M Oude Vrielink JA Bolijn A . Regulation of the adenomatous polyposis coli gene by the miR-135 family in colorectal cancer. Cancer Res. (2008) 68:5795–802. doi: 10.1158/0008-5472.CAN-08-0951, PMID: 18632633

[201] Yang J Ma D Fesler A Zhai H Leamniramit A Li W . Expression analysis of microRNA as prognostic biomarkers in colorectal cancer. Oncotarget. (2017) 8:52403. doi: 10.18632/oncotarget.14175, PMID: 28881738 PMC5581037

[202] Nijhuis A Thompson H Adam J Parker A Gammon L Lewis A . Remodelling of microRNAs in colorectal cancer by hypoxia alters metabolism profiles and 5-fluorouracil resistance. Hum Mol Genet. (2017) 26:1552–64. doi: 10.1093/hmg/ddx059, PMID: 28207045 PMC5393147

[203] Thulasingam S Massilamany C Gangaplara A Dai H Yarbaeva S Subramaniam S . miR-27b*, an oxidative stress-responsive microRNA modulates nuclear factor-kB pathway in RAW 264.7 cells. Mol Cell Biochem. (2011) 352:181–8. doi: 10.1007/s11010-011-0752-2, PMID: 21350856

[204] Wallace L Aikhionbare K Banerjee S Peagler K Pitts M Yao X . Differential expression profiles of mitogenome associated microRNAs among colorectal adenomatous polyps. Cancer Res J. (2021) 9:23. doi: 10.11648/j.crj.20210901.14, PMID: 33628862 PMC7899164

[205] Shaughnessy DT McAllister K Worth L Haugen AC Meyer JN Domann FE . Mitochondria, energetics, epigenetics, and cellular responses to stress. Environ Health Perspect. (2014) 122:1271–8. doi: 10.1289/ehp.1408418, PMID: 25127496 PMC4256704

[206] Adams J Mehrabi S Vatcharapijarn Y Iyamu OI Akwe JA Grizzle WE . Frequencies of mtDNA mutations in primary tissue of colorectal adenopolyps. Front Bioscience (Elite edition). (2013) 5:809., PMID: 23747897 10.2741/e661 PMC3739296

[207] Aikhionbare FO Khan M Carey D Okoli J Go R . Is cumulative frequency of mitochondrial DNA variants a biomarker for colorectal tumor progression? Mol Cancer. (2004) 3:1–4., PMID: 15482594 10.1186/1476-4598-3-30 PMC526214

[208] Clancy C Joyce MR Kerin MJ . The use of circulating microRNAs as diagnostic biomarkers in colorectal cancer. Cancer Biomarkers. (2015) 15:103–13. doi: 10.3233/CBM-140456, PMID: 25547322 PMC12928519

[209] Eslamizadeh S Heidari M Agah S Faghihloo E Ghazi H Mirzaei A . The role of microRNA signature as diagnostic biomarkers in different clinical stages of colorectal cancer. Cell J (Yakhteh). (2018) 20:220., PMID: 29633600 10.22074/cellj.2018.5366 PMC5893294

[210] Raisch J Darfeuille-Michaud A Nguyen HTT . Role of microRNAs in the immune system, inflammation and cancer. World J Gastroenterology: WJG. (2013) 19:2985. doi: 10.3748/wjg.v19.i20.2985, PMID: 23716978 PMC3662938

[211] Zeng M Zhu L Li L Kang C . miR-378 suppresses the proliferation, migration and invasion of colon cancer cells by inhibiting SDAD1. Cell Mol Biol Lett. (2017) 22:1–13. doi: 10.1186/s11658-017-0041-5, PMID: 28725241 PMC5514464

[212] Tong H Zhang L Gao J Wen S Zhou H Feng S . Methylation of mitochondrial DNA displacement loop region regulates mitochondrial copy number in colorectal cancer. Mol Med Rep. (2017) 16:5347–53. doi: 10.3892/mmr.2017.7264, PMID: 28849075 PMC5647067

[213] Diesch J Zwick A Garz A-K Palau A Buschbeck M Götze KS . A clinical-molecular update on azanucleoside-based therapy for the treatment of hematologic cancers. Clin Epigenet. (2016) 8:1–11. doi: 10.1186/s13148-016-0237-y, PMID: 27330573 PMC4915187

[214] Schneider-Stock R Diab-Assef M Rohrbeck A Foltzer-Jourdainne C Boltze C Hartig R . RETRACTION: 5-aza-Cytidine is a potent inhibitor of DNA methyltransferase 3a and induces apoptosis in HCT-116 colon cancer cells via Gadd45-and p53-dependent mechanisms. J Pharmacol Exp Ther. (2005) 312:525–36. doi: 10.1124/jpet.104.074195, PMID: 15547111

[215] Hsi LC Xi X Wu Y Lippman SM . The methyltransferase inhibitor 5-aza-2-deoxycytidine induces apoptosis via induction of 15-lipoxygenase-1 in colorectal cancer cells. Mol Cancer Ther. (2005) 4:1740–6. doi: 10.1158/1535-7163.MCT-05-0218, PMID: 16275995

[216] Deng T Zhang Y . 5-Aza-2ng-1746.r tdine reactivates expression of RUNX3 by deletion of DNA methyltransferases leading to caspase independent apoptosis in colorectal cancer Lovo cells. Biomedicine Pharmacotherapy. (2009) 63:492–500. doi: 10.1016/j.biopha.2008.08.013, PMID: 18848767

[217] Xiong H Chen ZF Liang QC Du W Chen HM Su WY . Inhibition of DNA methyltransferase induces G2 cell cycle arrest and apoptosis in human colorectal cancer cells via inhibition of JAK2/STAT3/STAT5 signalling. J Cell Mol Med. (2009) 13:3668–79. doi: 10.1111/j.1582-4934.2009.00661.x, PMID: 20196786 PMC4516515

[218] Mossman D Kim K-T Scott RJ . Demethylation by 5-aza-2'-deoxycytidine in colorectal cancer cells targets genomic DNA whilst promoter CpG island methylation persists. BMC Cancer. (2010) 10:1–10. doi: 10.1186/1471-2407-10-366, PMID: 20618997 PMC2912869

[219] Flis S Gnyszka A Misiewicz-Krzemińska I Spławiński J . Decytabine enhances cytotoxicity induced by oxaliplatin and 5-fluorouracil in the colorectal cancer cell line Colo-205. Cancer Cell Int. (2009) 9:1–10. doi: 10.1186/1475-2867-9-10, PMID: 19397792 PMC2683807

[220] Yu M Shi Y Wei X Yang Y Zhou Y Hao X . Depletion of mitochondrial DNA by ethidium bromide treatment inhibits the proliferation and tumorigenesis of T47D human breast cancer cells. Toxicol Lett. (2007) 170:83–93. doi: 10.1016/j.toxlet.2007.02.013, PMID: 17391873

[221] Dickinson A Yeung K Donoghue J Baker M Kelly RD McKenzie M . The regulation of mitochondrial DNA copy number in glioblastoma cells. Cell Death Differentiation. (2013) 20:1644–53. doi: 10.1038/cdd.2013.115, PMID: 23995230 PMC3824586

[222] Mei H Sun S Bai Y Chen Y Chai R Li H . Reduced mtDNA copy number increases the sensitivity of tumor cells to chemotherapeutic drugs. Cell Death Dis. (2015) 6:e1710–0. doi: 10.1038/cddis.2015.78, PMID: 25837486 PMC4650546

[223] Trinei M Berniakovich I Pelicci PG Giorgio M . Mitochondrial DNA copy number is regulated by cellular proliferation: A role for Ras and p66Shc. Biochim Biophys Acta (BBA)-Bioenergetics. (2006) 1757:624–30. doi: 10.1016/j.bbabio.2006.05.029, PMID: 16829231

[224] Yan Z Yuan Q He Y Peng F Liu Y Zhang H . Mitochondrial DNA haplogroup M7: a predictor of poor prognosis for colorectal cancer patients in Chinese population. Cancer Sci. (2023) 114:1056–66. doi: 10.1111/cas.v114.3, PMID: 36382493 PMC9986060

[225] Ferreira A Serafim TL Sardao VA Cunha OMRAT . Role of mt DNAeaomra10 mitoepigenetic phenomena in cancer. Eur J Clin Invest. (2015) 45:44–9. doi: 10.1111/eci.2014.45.issue-s1 25524586

[226] Kukat C Davies KM Wurm CA Spåhr H Bonekamp NA Kühl I . Cross-strand binding of TFAM to a single mtDNA molecule forms the mitochondrial nucleoid. Proc Natl Acad Sci. (2015) 112:11288–93. doi: 10.1073/pnas.1512131112, PMID: 26305956 PMC4568684

[227] Gao J Wen S Zhou H Feng S . De-methylation of displacement loop of mitochondrial DNA is associated with increased mitochondrial copy number and nicotinamide adenine dinucleotide subunit 2 expression in colorectal cancer. Mol Med Rep. (2015) 12:7033–8. doi: 10.3892/mmr.2015.4256, PMID: 26323487

[228] Rebelo AP Williams SL Moraes CT . *In vivo* methylation of mtDNA reveals the dynamics of protein–mtDNA interactions. Nucleic Acids Res. (2009) 37:6701–15. doi: 10.1093/nar/gkp727, PMID: 19740762 PMC2777446

[229] Lei T Rui Y Xiaoshuang Z Jinglan Z Jihong Z . Mitochondria transcription and cancer. Cell Death Discov. (2024) 10:168. doi: 10.1038/s41420-024-01926-3, PMID: 38589371 PMC11001877

[230] Zhu Y Xu J Hu W Wang F Zhou Y Xu W . TFAM depletion overcomes hepatocellular carcinoma resistance to doxorubicin and sorafenib through AMPK activation and mitochondrial dysfunction. Gene. (2020) 753:144807. doi: 10.1016/j.gene.2020.144807, PMID: 32461017

[231] Weinberg F Hamanaka R Wheaton WW Weinberg S Joseph J Lopez M . Mitochondrial metabolism and ROS generation are essential for Kras-mediated tumorigenicity. Proc Natl Acad Sci. (2010) 107:8788–93. doi: 10.1073/pnas.1003428107, PMID: 20421486 PMC2889315

[232] Zhao Y Wang Y Zhao J Zhang Z Jin M Zhou F . PDE2 inhibits PKA-mediated phosphorylation of TFAM to promote mitochondrial Ca2+-induced colorectal cancer growth. Front Oncol. (2021) 11:663778. doi: 10.3389/fonc.2021.663778, PMID: 34235078 PMC8256694

[233] Liu Y Jin M Wang Y Zhu J Tan R Zhao J . MCU-induced mitochondrial calcium uptake promotes mitochondrial biogenesis and colorectal cancer growth. Signal Transduction Targeted Ther. (2020) 5:59. doi: 10.1038/s41392-020-0155-5, PMID: 32371956 PMC7200750

[234] Li Y Yang Q Chen H Yang X Han J Yao X . TFAM downregulation promotes autophagy and ESCC survival through mtDNA stress-mediated STING pathway. Oncogene. (2022) 41:3735–46. doi: 10.1038/s41388-022-02365-z, PMID: 35750756

[235] Lemnrau A Brook MN Fletcher O Coulson P Tomczyk K Jones M . Mitochondrial DNA copy number in peripheral blood cells and risk of developing breast cancer. Cancer Res. (2015) 75:2844–50. doi: 10.1158/0008-5472.CAN-14-1692, PMID: 25977328

[236] Ye X Han Y Zhang L Liu W Zuo J . MTERF4 regulates the mitochondrial dysfunction induced by MPP+ in SH-SY5Y cells. Biochem Biophys Res Commun. (2015) 464:214–20. doi: 10.1016/j.bbrc.2015.06.119, PMID: 26102036

[237] Wang X-L Liu Q Chen G-J Li M-L Ding Y-H . Overexpression of MTERF4 promotes the amyloidogenic processing of APP by inhibiting ADAM10. Biochem Biophys Res Commun. (2017) 482:928–34. doi: 10.1016/j.bbrc.2016.11.135, PMID: 27894840

[238] Han Y Gao P Qiu S Zhang L Yang L Zuo J . MTERF2 contributes to MPP+-induced mitochondrial dysfunction and cell damage. Biochem Biophys Res Commun. (2016) 471:177–83. doi: 10.1016/j.bbrc.2016.01.156, PMID: 26826381

[239] Chen G Dai J Tan S Meng S Liu Z Li M . MTERF1 regulates the oxidative phosphorylation activity and cell proliferation in HeLa cells. Acta Biochim Biophys Sin. (2014) 46:512–21. doi: 10.1093/abbs/gmu029, PMID: 24777141

[240] Liu Q Zhang L Zou Y Tao Y Wang B Li B . Modulating p-AMPK/mTOR pathway of mitochondrial dysfunction caused by MTERF1 abnormal expression in colorectal cancer cells. Int J Mol Sci. (2022) 23:12354. doi: 10.3390/ijms232012354, PMID: 36293209 PMC9604058

[241] Zi J Wang W Sun M Mei W Li S Li B . A high expression of MTERF3 correlates with tumor progression and predicts poor outcomes in patients with brain glioma. Int J Clin Exp Pathol. (2019) 12:1909., PMID: 31934014 PMC6947131

[242] Xiong W Luo Y Zhang C Tan D Zuo S . Expression, purification of recombinant human mitochondrial transcription termination factor 3 (hMTERF3) and preparation of polyclonal antibody against hMTERF3. Appl Biochem Biotechnol. (2012) 167:2318–29. doi: 10.1007/s12010-012-9754-0, PMID: 22711491

[243] Liu X Cao X Liu C Cao Y Zhao Q Tan X . MTERFD1 promotes cell growth and irradiation resistance in colorectal cancer by upregulating interleukin-6 and interleukin-11. Int J Biol Sci. (2019) 15:2750. doi: 10.7150/ijbs.36916, PMID: 31754344 PMC6854380

[244] Choi Y Park S Yang S Kim E Cho Y Yang H-J . Identifying TP53RK as a key regulator of colorectal cancer survival and a potential therapeutic target. Sci Rep. (2025) 15:36122. doi: 10.1038/s41598-025-21082-7, PMID: 41102525 PMC12533122

[245] Zhang Y Han T Zhang H Li J Chu J Liu J . Immune characteristics and SALL1 methylation as prognostic biomarkers in primary and metastasis colorectal cancer. Sci Rep. (2025) 15:28292. doi: 10.1038/s41598-025-13191-0, PMID: 40754617 PMC12319110

[246] Chen J Li Z-Y Zheng G Cao L Guo Y-M Lian Q . RNF4 mediated degradation of PDHA1 promotes colorectal cancer metabolism and metastasis. NPJ Precis Oncol. (2024) 8:258. doi: 10.1038/s41698-024-00724-5, PMID: 39521913 PMC11550450

[247] Luo C Xiao Z Yang W . GNG2 inhibits brain metastases from colorectal cancer via PI3K/AKT/mTOR signaling pathway. Sci Rep. (2025) 15:1787. doi: 10.1038/s41598-025-85592-0, PMID: 39805936 PMC11730682

[248] Luo X-R Huang L-Z Yin J Xiong Z-M Li W-X Liao C . FSTL3 promotes colorectal cancer by activating the HIF1 pathway. Gene. (2025) 954:149435. doi: 10.1016/j.gene.2025.149435, PMID: 40154584

[249] Pang L Zhou F Liu Y Ali H Khan F Heimberger AB . Epigenetic regulation of tumor immunity. J Clin Invest. (2024) 134:e178540. doi: 10.1172/JCI178540, PMID: 39133578 PMC11178542

[250] Dang T Guan X Cui L Ruan Y Chen Z Zou H . Epigenetics and immunotherapy in colorectal cancer: progress and promise. Clin Epigenet. (2024) 16:123. doi: 10.1186/s13148-024-01740-9, PMID: 39252116 PMC11385519

[251] Yang J Xu J Wang W Zhang B Yu X Shi S . Epigenetic regulation in the tumor microenvironment: molecular mechanisms and therapeutic targets. Signal Transduction Targeted Ther. (2023) 8:210. doi: 10.1038/s41392-023-01480-x, PMID: 37217462 PMC10203321

[252] Ji Y Xiao C Fan T Deng Z Wang D Cai W . The epigenetic hallmarks of immune cells in cancer. Mol Cancer. (2025) 24:66. doi: 10.1186/s12943-025-02255-4, PMID: 40038722 PMC11881328

[253] Guven DC Kavgaci G Erul E Syed MP Magge T Saeed A . The efficacy of immune checkpoint inhibitors in microsatellite stable colorectal cancer: a systematic review. Oncologist. (2024) 29:e580–600. doi: 10.1093/oncolo/oyae013, PMID: 38309719 PMC11067816

[254] Wang Z Liu Y Wang K Ma L . Efficacy and safety of PD-1 and PD-L1 inhibitors in advanced colorectal cancer: a meta-analysis of randomized controlled trials. BMC Gastroenterol. (2024) 24:461. doi: 10.1186/s12876-024-03554-8, PMID: 39696009 PMC11658155

[255] Han Y-J Shao C-Y Yao Y Zhang Z Fang M-Z Gong T . Immunotherapy of microsatellite stable colorectal cancer: resistance mechanisms and treatment strategies. Postgraduate Med J. (2024) 100:373–81. doi: 10.1093/postmj/qgad136, PMID: 38211949

[256] Polak R Zhang ET Kuo CJ . Cancer organoids 2.0: modelling the complexity of the tumour immune microenvironment. Nat Rev Cancer. (2024) 24:523–39. doi: 10.1038/s41568-024-00706-6, PMID: 38977835

[257] Steinert EM Vasan K Chandel NS . Mitochondrial metabolism regulation of T cell–mediated immunity. Annu Rev Immunol. (2021) 39:395–416. doi: 10.1146/annurev-immunol-101819-082015, PMID: 33902315 PMC10403253

[258] Kuo C-L Lin Y-C Lo YK Lu Y-Z Babuharisankar AP Lien H-W . The mitochondrial stress signaling tunes immunity from a view of systemic tumor microenvironment and ecosystem. Iscience. (2024) 27:110710. doi: 10.1016/j.isci.2024.110710, PMID: 39262792 PMC11388186

[259] Monson KR Ferguson R Handzlik JE Morales L Xiong J Chat V . Inherited mitochondrial genetics as a predictor of immune checkpoint inhibition efficacy in melanoma. Nat Med. (2025) 31:2385–96. doi: 10.1038/s41591-025-03699-3, PMID: 40473950 PMC12283385

[260] Yang M-Q Zhang S-L Sun L Huang L-T Yu J Zhang J-H . Targeting mitochondria: restoring the antitumor efficacy of exhausted T cells. Mol Cancer. (2024) 23:260. doi: 10.1186/s12943-024-02175-9, PMID: 39563438 PMC11575104

[261] Pandey S Anang V Schumacher MM . Mitochondria driven innate immune signaling and inflammation in cancer growth, immune evasion, and therapeutic resistance. Int Rev Cell Mol Biol. (2024) 386:223–47., PMID: 38782500 10.1016/bs.ircmb.2024.01.006

[262] Pinto De Almeida NJ Meid ÁJ Hong R Rajeh A Nogueira F Szadai L . Mitochondrial dysfunction and immune suppression in BRAF V600Eessional metastatic melanoma. Clin Trans Med. (2024) 14:e1773., PMID: 39032005 10.1002/ctm2.1773 PMC11259597

[263] Wimalasena VK Wang T Sigua LH Durbin AD Qi J . Using chemical epigenetics to target cancer. Mol Cell. (2020) 78:1086–95. doi: 10.1016/j.molcel.2020.04.023, PMID: 32407673 PMC8033568

[264] Allfrey VG Faulkner R Mirsky A . Acetylation and methylation of histones and their possible role in the regulation of RNA synthesis. Proc Natl Acad Sci. (1964) 51:786–94. doi: 10.1073/pnas.51.5.786, PMID: 14172992 PMC300163

[265] Bradner JE Hnisz D Young RA . Transcriptional addiction in cancer. Cell. (2017) 168:629–43. doi: 10.1016/j.cell.2016.12.013, PMID: 28187285 PMC5308559

[266] Dancy BM Cole PA . Protein lysine acetylation by p300/CBP. Chem Rev. (2015) 115:2419–52. doi: 10.1021/cr500452k, PMID: 25594381 PMC4378506

[267] Lasko LM Jakob CG Edalji RP Qiu W Montgomery D Digiammarino EL . Discovery of a selective catalytic p300/CBP inhibitor that targets lineage-specific tumours. Nature. (2017) 550:128–32. doi: 10.1038/nature24028, PMID: 28953875 PMC6050590

[268] Weinert BT Narita T Satpathy S Srinivasan B Hansen BK Schölz C . Time-resolved analysis reveals rapid dynamics and broad scope of the CBP/p300 acetylome. Cell. (2018) 174:231–244.e212. doi: 10.1016/j.cell.2018.04.033, PMID: 29804834 PMC6078418

[269] Michaelides MR Kluge A Patane M Van Drie JH Wang C Hansen TM . Discovery of spiro oxazolidinediones as selective, orally bioavailable inhibitors of p300/CBP histone acetyltransferases. ACS Medicinal Chem Lett. (2018) 9:28–33. doi: 10.1021/acsmedchemlett.7b00395, PMID: 29348807 PMC5767893

[270] Zucconi BE Makofske JL Meyers DJ Hwang Y Wu M Kuroda MI . Combination targeting of the bromodomain and acetyltransferase active site of p300/CBP. Biochemistry. (2019) 58:2133–43. doi: 10.1021/acs.biochem.9b00160, PMID: 30924641 PMC6948846

[271] Sen P Lan Y Li CY Sidoli S Donahue G Dou Z . Histone acetyltransferase p300 induces *de novo* super-enhancers to drive cellular senescence. Mol Cell. (2019) 73:684–698.e688. doi: 10.1016/j.molcel.2019.01.021, PMID: 30773298 PMC6688479

[272] Ramos YF Hestand MS Verlaan M Krabbendam E Ariyurek Y van Galen M . Genome-wide assessment of differential roles for p300 and CBP in transcription regulation. Nucleic Acids Res. (2010) 38:5396–408. doi: 10.1093/nar/gkq184, PMID: 20435671 PMC2938195

[273] Jia Y-L Xu M Dou C-W Liu Z-K Xue Y-M Yao B-W . P300/CBP-associated factor (PCAF) inhibits the growth of hepatocellular carcinoma by promoting cell autophagy. Cell Death Dis. (2016) 7:e2400–0. doi: 10.1038/cddis.2016.247, PMID: 27711074 PMC5133959

[274] Malatesta M Steinhauer C Mohammad F Pandey DP Squatrito M Helin K . Histone acetyltransferase PCAF is required for hedgehog–gli-dependent transcription and cancer cell proliferation. Cancer Res. (2013) 73:6323–33. doi: 10.1158/0008-5472.CAN-12-4660, PMID: 23943798

[275] Daigle SR Olhava EJ Therkelsen CA Majer CR Sneeringer CJ Song J . Selective killing of mixed lineage leukemia cells by a potent small-molecule DOT1L inhibitor. Cancer Cell. (2011) 20:53–65. doi: 10.1016/j.ccr.2011.06.009, PMID: 21741596 PMC4046888

[276] McCabe MT Ott HM Ganji G Korenchuk S Thompson C Van Aller GS . EZH2 inhibition as a therapeutic strategy for lymphoma with EZH2-activating mutations. Nature. (2012) 492:108–12. doi: 10.1038/nature11606, PMID: 23051747

[277] Zhang H Qi J Reyes JM Li L Rao PK Li F . Oncogenic deregulation of EZH2 as an opportunity for targeted therapy in lung cancer. Cancer Discov. (2016) 6:1006–21. doi: 10.1158/2159-8290.CD-16-0164, PMID: 27312177 PMC5010480

[278] Mohammad F Weissmann S Leblanc B Pandey DP Højfeldt JW Comet I . EZH2 is a potential therapeutic target for H3K27M-mutant pediatric gliomas. Nat Med. (2017) 23:483–92. doi: 10.1038/nm.4293, PMID: 28263309

[279] Huang X Yan J Zhang M Wang Y Chen Y Fu X . Zhang X: Targeting epigenetic crosstalk as a therapeutic strategy for EZH2-aberrant solid tumors. Cell. (2018) 175:186–199.e119., PMID: 30220457 10.1016/j.cell.2018.08.058

[280] Tarighat SS Santhanam R Frankhouser D Radomska HS Lai H Anghelina M . The dual epigenetic role of PRMT5 in acute myeloid leukemia: gene activation and repression via histone arginine methylation. Leukemia. (2016) 30:789–99. doi: 10.1038/leu.2015.308, PMID: 26536822 PMC8034866

[281] Farrelly LA Thompson RE Zhao S Lepack AE Lyu Y Bhanu NV . Histone serotonylation is a permissive modification that enhances TFIID binding to H3K4me3. Nature. (2019) 567:535–9. doi: 10.1038/s41586-019-1024-7, PMID: 30867594 PMC6557285

[282] Jain PG Patel BD . Medicinal chemistry approaches of poly ADP-Ribose polymerase 1 (PARP1) inhibitors as anticancer agents-A recent update. Eur J Medicinal Chem. (2019) 165:198–215. doi: 10.1016/j.ejmech.2019.01.024, PMID: 30684797

[283] Malinee M Pandian GN Sugiyama H . Targeted epigenetic induction of mitochondrial biogenesis enhances antitumor immunity in mouse model. Cell Chem Biol. (2022) 29:463–475.e466. doi: 10.1016/j.chembiol.2021.08.001, PMID: 34520746

[284] Lei Y Han P Chen Y Wang H Wang S Wang M . Protein arginine methyltransferase 3 promotes glycolysis and hepatocellular carcinoma growth by enhancing arginine methylation of lactate dehydrogenase A. Clin Trans Med. (2022) 12:e686. doi: 10.1002/ctm2.v12.1, PMID: 35090076 PMC8797063

[285] Zhang T Gong Y Meng H Li C Xue L . Symphony of epigenetic and metabolic regulation—interaction between the histone methyltransferase EZH2 and metabolism of tumor. Clin Epigenet. (2020) 12:72. doi: 10.1186/s13148-020-00862-0, PMID: 32448308 PMC7245796

[286] Jarrold J Davies CC . PRMTs and arginine methylation: cancer’s best-kept secret? Trends Mol Med. (2019) 25:993–1009., PMID: 31230909 10.1016/j.molmed.2019.05.007

[287] Morera L Lübbert M Jung M . Targeting histone methyltransferases and demethylases in clinical trials for cancer therapy. Clin Epigenet. (2016) 8:1–16. doi: 10.1186/s13148-016-0223-4, PMID: 27222667 PMC4877953

