# Supplementary material for: Targeting the epigenome and tumor heterogeneity: advances in immunotherapy for chemoresistant metastatic colorectal cancer
Source: Front Immunol. 2025 Dec 1;16:1623117. doi: 10.3389/fimmu.2025.1623117 (PMC12702937; doi:10.3389/fimmu.2025.1623117)
Supplement: Supplementary file 1 [file Presentation1.pptx]

## Slide 1
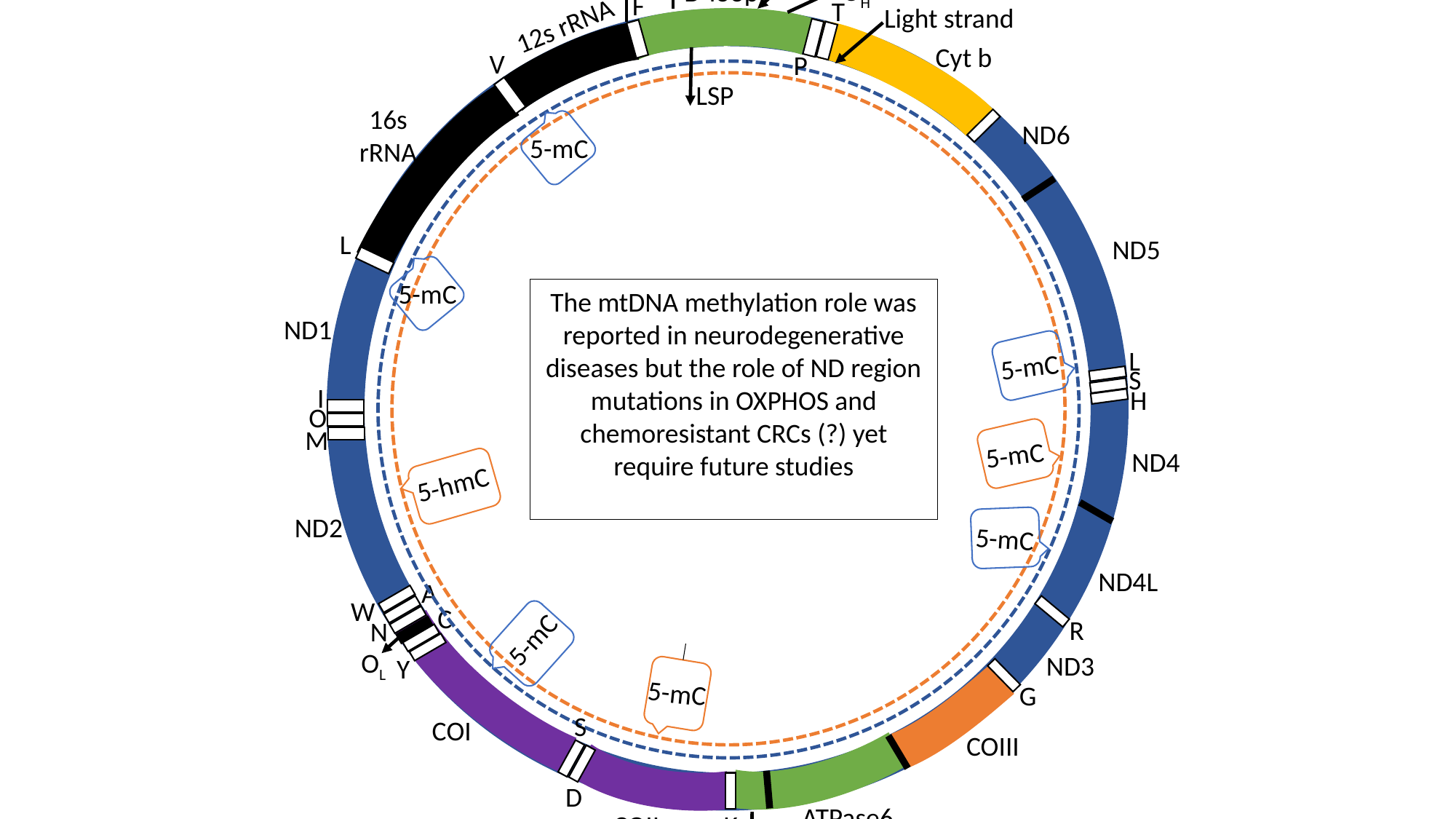

HSP1
HSP2
Heavy strand
OH
D-loop
F
T
Light strand
12s rRNA
Cyt b
V
P
LSP
16s
rRNA
ND6
5-mC
L
ND5
5-mC
ND1
5-mC
L
S
I
H
O
5-mC
M
5-hmC
ND4
5-mC
ND2
ND4L
A
W
C
5-mC
R
N
OL
ND3
Y
5-mC
G
S
COI
COIII
D
ATPase6
COII
K
ATPase8
The mtDNA methylation role was reported in neurodegenerative diseases but the role of ND region mutations in OXPHOS and chemoresistant CRCs (?) yet require future studies

## Slide 2
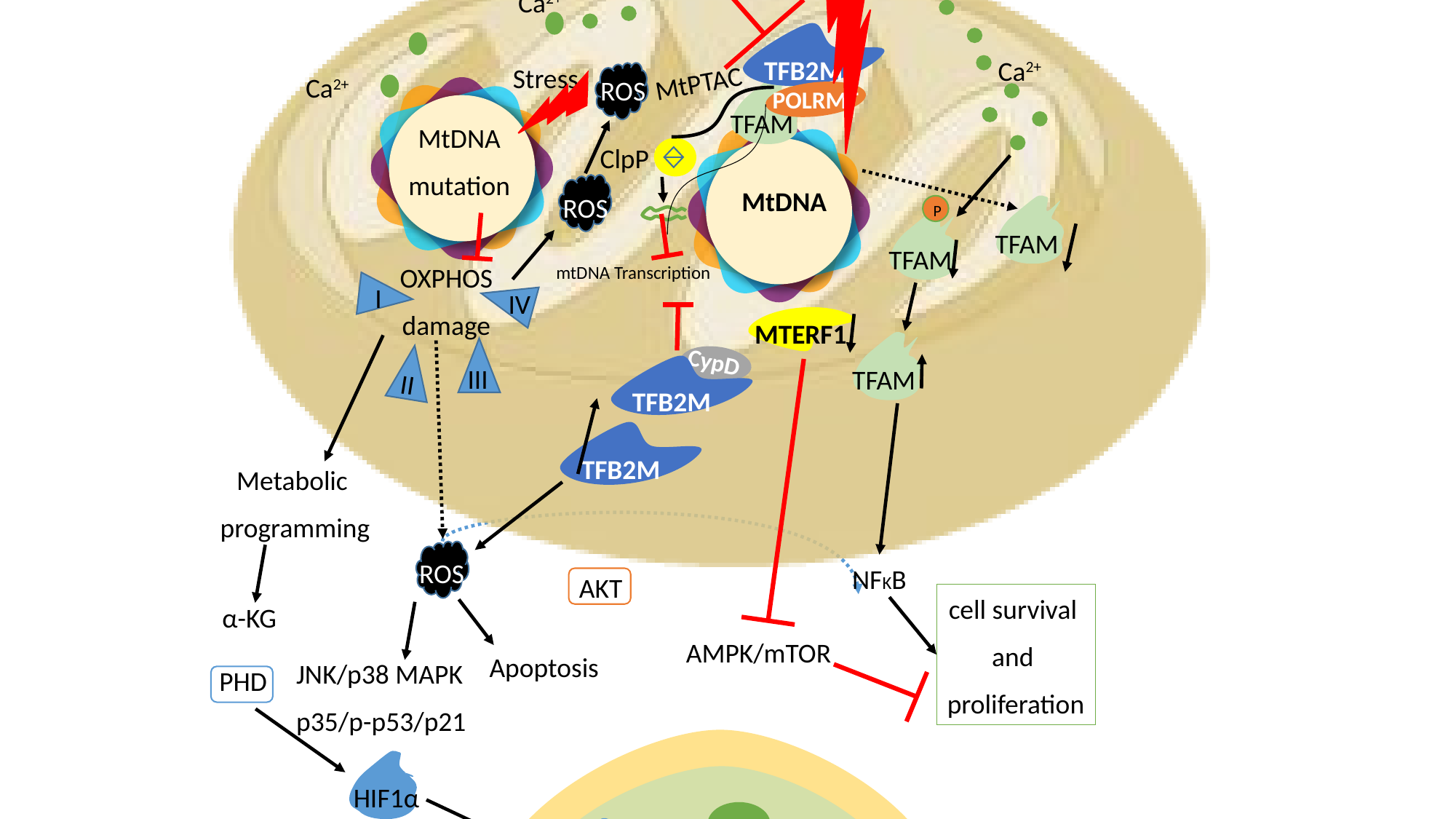

Novel molecules are required to block mitochondrial transcription, inhibiting mitochondrial OXPHOS in chemoresistant CRC cells
Ca2+
TFB2M
TFAM
POLRMT
Ca2+
Stress
MtPTAC
Ca2+
ROS
MtDNA
mutation
ClpP
MtDNA
ROS
P
TFAM
TFAM
OXPHOS
damage
mtDNA Transcription
IV
I
MTERF1
TFAM
III
CypD
TFB2M
II
TFB2M
Metabolic
programming
ROS
NFKB
AKT
cell survival
and
proliferation
α-KG
AMPK/mTOR
Apoptosis
JNK/p38 MAPK
p35/p-p53/p21
PHD
HIF1α
FBP2
c-Myc
HIF1α
+
β-catenin
+
TFAM
Cancer stem
cell signalling in chemo-resistant CRC
